# Multi-level association rule mining and network pharmacology to identify the polypharmacological effects of herbal materials and compounds in traditional medicine

**DOI:** 10.1093/bib/bbaf328

**Published:** 2025-07-07

**Authors:** Hyejin Yu, Kwanyong Choi, Ji Yeon Kim, Sunyong Yoo

**Affiliations:** Department of Intelligent Electronics and Computer Engineering, Chonnam National University, 77 Yongbong-ro, Buk-gu, Gwangju 61186, Republic of Korea; Department of Food Science and Biotechnology, Seoul National University of Science and Technology, 232 Gongneung-ro, Nowon-gu, Seoul 01811, Republic of Korea; Department of Food Science and Biotechnology, Seoul National University of Science and Technology, 232 Gongneung-ro, Nowon-gu, Seoul 01811, Republic of Korea; Department of Intelligent Electronics and Computer Engineering, Chonnam National University, 77 Yongbong-ro, Buk-gu, Gwangju 61186, Republic of Korea

**Keywords:** traditional medicine, herbal material, polypharmacology, combination therapy, association rule, network pharmacology

## Abstract

Many cultures worldwide have widely used traditional medicine (TM) to prevent or treat diseases. Herbal materials and their compounds used in TM offer many advantages for drug discovery, including cost-effectiveness, fewer side effects, and improved metabolism. However, the multi-compound and multi-target characteristics of TM prescriptions complicate drug discovery; meanwhile, previous studies have been limited by a lack of high-quality data, complex interpretation, and/or narrow analytical ranges. Thus, this study proposed a framework to identify potential therapeutic combinations of herbal materials and their compounds currently used in TM by integrating association rule mining (ARM) and network pharmacology analysis across multiple TM and biological levels. Subsequently, we collected prescriptions, herbal materials, compounds, genes, phenotypes, and all ensuing interactions to identify effective combinations of herbal materials and compounds using ARM for various symptoms and diseases. This proposed analytical approach was also applied to screen effective herbal material combinations and compounds for five phenotypes: asthma, diabetes, arthritis, stroke, and inflammation. The potential pharmacological effects of the inferred candidates were identified at the molecular level using structural network analysis and a literature review. In addition, compounds from *Morus alba*, *Ephedra sinica*, *Perilla frutescens*, and *Pinellia ternata*, which were strongly associated with asthma, were validated *in vitro*. Collectively, our study provides ethnopharmacological and biological evidence for the polypharmacological effects of herbal materials and their compounds, thus enhancing the understanding of the mechanisms involved in TM and suggesting potential candidates for prescriptions, dietary supplements, and drug combinations. The source code and results are available at https://github.com/bmil-jnu/InPETM.

## Introduction

Traditional medicine (TM), the world’s oldest form of health care, is widely used worldwide and remains a significant part of modern medicine [1–3]. TM generally employs herbal materials with compounds that modulate multiple target proteins. Moreover, the compounds used in TM typically have fewer side effects than synthetic drugs and are easily metabolized in the body due to the presence of numerous P450 enzymes with high activity [4, 5]. In addition, TM can be used to identify novel therapeutic uses for medicinal compounds based on ethnopharmacological data accumulated over thousands of years, thus reducing drug discovery costs [6, 7].

However, despite the increasing global use of TM, a lack of high-quality research validating its effectiveness remains, with many studies employing limited research approaches [8–10]. Moreover, information on the patterns of herbal material combinations in TM for treating diseases or symptoms has not been systematically documented within a unified and standardized framework [9, 11]. The complicated multi-compound and multi-target characteristics of TM prescriptions and herbal materials also make understanding the polypharmacological mechanisms involved in the biological interactions between the components and TM targets difficult [12–17]. Additionally, manually collecting information from large TM databases on the pharmacological effects of herbal materials and their compounds using traditional methods such as *in vivo* or *in vitro* screening is inefficient and time consuming [11, 18].

Various *in silico* approaches, such as ligand- or structure-based methods, have been proposed to predict drug candidates using herbal materials or compounds with polypharmacological profiles to address these limitations. Ligand-based methods rely primarily on the properties of known ligands, while structure-based methods depend on large volumes of high-quality structural data, both of which limit accurate predictions [19, 20]. In addition, machine learning–based strategies employ algorithms such as logistic regression, random forest, support vector machine, and neural network models for drug repositioning in TM [21–24]. However, most previous studies employing these approaches have focused only on specific diseases or compounds and/or relied on relatively small datasets; meanwhile, many machine models are difficult to interpret because of their black-box nature.

Therefore, the present study proposed an integrated analysis using association rule mining (ARM) and network pharmacology to identify potentially useful therapeutic combinations of herbal materials and compounds in TM. Indeed, ARM represents a potential solution to the limitations of machine-learning approaches, which can be used to process large amounts of data with high interpretability through its rule-based approach [11, 25]. While some previous studies have used ARM together with network pharmacology models to identify new bioactive ingredients and to determine the prescribing mechanisms used in TM, these studies have generally only investigated potential combinations of herbal materials or compounds without validating their effectiveness through a systematic pipeline incorporating multiple levels of biological evidence [26–28]. In addition, to our knowledge, no integrated studies have combined TM and biological expertise to conduct a large-scale analysis that is not limited to specific diseases.

Therefore, this study proposed a highly interpretable analytical framework for determining the prescribing patterns for multiple diseases using large-scale TM data. Hence, in this study, we collected diverse data on TM prescriptions, herbal materials, compounds, target proteins, diseases, and their relationships. Based on these data, ARM was used to identify candidate herbal material combinations or compounds with potential activity against diseases; meanwhile, network pharmacology was employed to provide supporting evidence for the selected candidates at the molecular level. A literature review of prior *in vitro* experiments was conducted to validate the predicted herbal material combinations and compounds. Thus, the proposed approach in this study can be employed to advance drug discovery and translational research alongside TM by providing mechanistic evidence for the potential pharmacological effects of candidates and associated biological pathways.

## Materials and methods

### Materials

We built a dataset of information on TM materials and relevant biological information using various databases. TM prescriptions, herbal materials, compounds, genes, phenotypes, and interaction information for Korea, China, and Japan were collected from the Korean Traditional Knowledge Portal (KTKP) [29], Traditional Chinese Medicine Information Database (TCM-ID) [30], KampoDB [31], and Compound Combination-Oriented Natural Product Database with Unified Terminology (COCONUT) [26]. Compound–phenotype and compound–gene associations were taken from the DrugBank [32], Therapeutic Target Database (TTD) [33], Comparative Toxicogenomics Database (CTD) [34], BindingDB [35], Search Tool for Interacting Chemicals (STITCH) [36], and FooDB [37]. Gene–phenotype associations were collected from the CTD, while herb–compound associations were collected from Collective Molecular Activities of Useful Plants (CMAUP) [38]. In total, 16 260 prescriptions, 7755 phenotypes, 5279 herbal materials, 43 454 compounds, and 19 103 genes were used. The number of data entries for each entity and relationship collected from the databases, along with details on the standardization methods employed, is provided in Supplementary Tables S1 and S2. Additionally, 26 771 nodes and 856 635 edges were collected from BioGRID v.4.4.218 to build a human protein interactome network [39].

### Method overview

**Figure 1:**
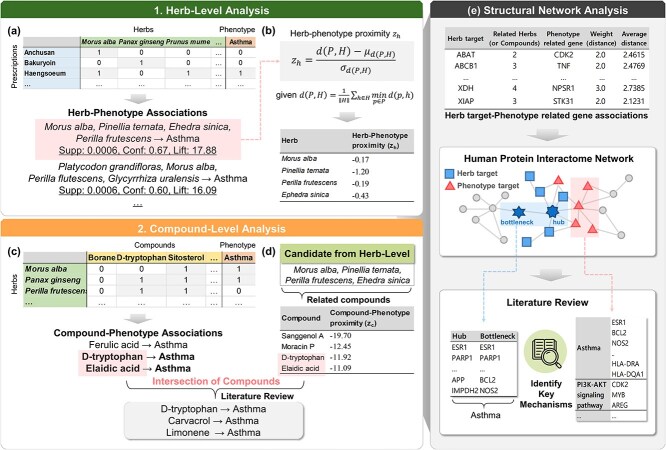
Overview of the integrated analysis pipeline used to identify therapeutic herbal material combinations and compounds in the present study. (a) ARM at the herb level was conducted to identify herbal material combinations, from which the candidates with the highest evaluation scores were selected. (b) Herb–phenotype proximities were calculated for the inferred herbal material combinations. (c) ARM at the compound level was employed to identify candidate compounds. (d) Compound–phenotype proximities for the compounds related to the inferred herbal material combinations were calculated. For compounds identified in both the compound–phenotype ARM and compound–phenotype network analyses, those known to be effective for a disease were determined based on a literature review. (e) Structural network analysis, including the literature review, was conducted by identifying key mechanisms of action (MoAs) for the inferred herbal material combinations and compounds in humans to validate the results.

The proposed integrative analysis framework was divided into two steps: (i) herb-level analysis, which identified potential therapeutic herbal material combinations and investigated their mechanisms within the human protein interactome network, and (ii) compound-level analysis, which explored potential therapeutic compounds and provided additional evidence by analyzing in detail those overlapping compounds that were found in both the herb- and compound-level analyses (Fig. 1).

### Herb-level analysis

#### Herb–phenotype association rule mining

The main hypothesis of the herb–phenotype ARM analysis was that, if certain herbal material combinations are frequently found in prescriptions for a specific phenotype, these combinations may have therapeutic potential for that phenotype. To test this hypothesis, we first created a transaction dataset containing herbal material combinations and the known phenotypes: these combinations were used to treat based on prescription information (Fig. 1a). We employed 16 260 prescriptions and 628 herbal materials for the herb-level analysis.

We then used the Apriori algorithm to explore frequent item sets in the transaction dataset and generate association rules [40, 41]. Generally, association rules possess the form “antecedent → consequent,” meaning that whenever an antecedent occurs, a consequent is also likely to occur [42]. Therefore, the association rules in the present study were “herb A, herb B, herb C, etc. → phenotype.”

We used three metrics to evaluate these association rules: support, confidence, and lift. The support metric refers to the frequency of occurring patterns, which can be expressed as the proportion of all transactions that contain both the antecedent and consequent [43] and the confidence metric is used to measure the implication strength, which is the ratio of transactions containing the antecedent to transactions containing the consequent. Only those rules that satisfy the minimum support and confidence can be considered valid. The lift value, which is the ratio of the confidence metric to the probability of occurrence for the consequent, is frequently used to measure the utility of the association rule [44]. A lift value <1 indicates a stronger association upon rule negation, meaning a negative correlation between the antecedent and the consequent.

We generated association rules for the frequent item sets that satisfied the minimum support value of 0.0005 (i.e. derived from more than 8 actual prescriptions among 16 260) for performing ARM on the large-scale TM database. We selected the highest confidence and lift values from these rules to identify the most relevant frequent item sets [41, 45].

#### Herb–phenotype network analysis

Network pharmacological analysis was employed to validate the association rules generated by the herb–phenotype ARM (Fig. 1b). Previous studies have shown that a compound is more likely to be associated with a phenotype when the target of the compound and phenotype-related genes are located close together in the human protein interactome network. These studies used the relative proximity (${z}_c$) as an unbiased measure to represent the relationship between the compound target and the phenotype-related genes in the network, capturing the statistical significance (*z*-score) of their calculated distance [26, 46]. Of the measures used to calculate the relative proximity, the closest distance metric, which is the average shortest path length between the compound targets and the nearest phenotype-related genes, provides the most accurate drug efficacy screening results. Similar results have been observed in herb–phenotype association analyses [47].

Thus, the relative proximity based on the closest distance was employed to quantify the association between the targets of the compounds in herbal materials (i.e. herb targets) and phenotype-related genes. If the herb target of each herbal material matches or is sufficiently close enough to the phenotype-related gene or genes, the herbal material combination would likely be associated with that phenotype.

We calculated herb–phenotype proximity ${z}_h$, i.e. the relative proximity between the herbal material and the phenotype within a combination using the antecedent of the selected association rule, using the following process:

(1) The distance to all genes in the phenotype-related gene group was calculated for each gene in the herb target group, and the phenotype-related gene closest to each herb target was determined.
(2) The closest distance was calculated by adding all of the distances for each herb target to the closest phenotype-related gene and calculating an average. Simply, given the phenotype-related gene group $P$, the herb target group $H$, and the shortest path length between nodes $p$ and$h$ in the network $d\left(p,h\right)$, the closest distance $d$ between $P$ and $H$ can be calculated using Equation (1):
  (1) \begin{equation*} \mathrm{\it d}\left(\mathrm{\it P},\mathrm{\it H}\right)=\frac{1}{\parallel H\parallel}\sum_{h\in H}{\min}_{p\in P}d\left(p,h\right)\end{equation*}
(3) genes from the human protein interactome network with the same degree of centrality were randomly selected to create two groups of the same size as the herb target group and the phenotype-related gene group, respectively, and the closest distance between them was identified.
(4) Point (4) was repeated 1000 times to obtain a reference distance distribution for the expected distances between two randomly selected gene groups.
(5) The mean ${\mu}_{d\left(P,H\right)}$ and SD ${\sigma}_{d\left(P,H\right)}$ of the reference distance distribution were computed to calculate the relative proximity ${z}_h$, as shown in Equation (2):
  (2) \begin{equation*} {\displaystyle \begin{array}{c}{\mathrm\mathit{z}}_{\mathrm{\it h}}=\frac{d\left(\mathrm{\it P},\mathrm{\it H}\right)-{\mu}_{d\left(\mathrm{\it P},\mathrm{\it H}\right)}}{\sigma_{d\left(\mathrm{\it P},\mathrm{\it H}\right)}}\end{array}} \end{equation*}
  we then compared the calculated proximity with a threshold defined in previous research because it is acknowledged to provide good coverage of known compound–phenotype associations and few false positives [26, 46, 48]. We considered the combination strongly related to the phenotype if most herbal materials had a proximity ${z}_h\le -0.15$.

### Compound-level analysis

#### Compound–phenotype association rule mining

We constructed a transaction dataset containing various compounds and phenotype information for the herbal materials to conduct the compound–phenotype ARM analysis (Fig. 1c). This dataset contained 5187 herbal materials and 43 454 compounds for the compound-level analysis. The association rule for this dataset was “compound → phenotype,” with a minimum support value of 0.0008 (i.e. derived from >5 actual prescriptions among 5187).

#### Compound–phenotype network analysis

To ensure that the herb-level and compound-level analyses yielded consistent results, we compared the compounds identified using the compound–phenotype ARM with the compounds associated with the inferred herbal material combinations and extracted those that overlapped (Fig. 1d).

We used the compound–phenotype proximity ${z}_c$, which is the relative proximity calculated between a compound and a phenotype. Given a compound target group $C$ and a phenotype-related gene group $P$, the shortest path length between nodes $p$ and $c$ in the network, $d\left(p,c\right)$, the closest distance between $P$ and $C$ is provided using Equation (3).


(3) \begin{align*} {\displaystyle \begin{array}{c}\mathrm{\it d}\left(\mathrm{\it P},\mathrm{\it C}\right)=\frac{1}{\parallel \mathrm{\it C}\parallel}\sum\limits_{c\in C}{\min}_{\mathrm{\it p}\in \mathrm{\it P}}d\left(p,c\right)\end{array}} \end{align*}

the relative proximity ${z}_c$ can be obtained using the reference distance distribution derived through iterative computation, as shown in Equation (4).


(4) \begin{equation*} {z}_c=\frac{d\left(P,C\right)-{\mu}_{d\left(P,C\right)}}{\sigma_{d\left(P,C\right)}} \end{equation*}

the calculated relative proximity was validated using the same criteria as for herb–phenotype proximity. To provide additional evidence, we conducted a compound–phenotype network analysis on the herbal material combination-related compounds to identify compounds proximal to the phenotype in the human protein interactome network. We then extracted compounds identified in the compound–phenotype network analysis and the compound–phenotype ARM. If the literature review revealed evidence that certain compounds in the overlap between the two analyses were effective against a disease, we concluded that combining herbal materials associated with these compounds would also be useful in treating that disease.

### Structural network analysis

We performed a structural network analysis at the end of each of the other analyses based on the association between biological targets (i.e. the herb or compound targets) and phenotype-related genes (Fig. 1e). This approach enables the influence of biological targets on phenotype-associated mechanisms to be investigated in humans [49]. To identify important biological targets, we used network topological features to identify hubs and bottlenecks, which tend to be important targets in networks involved in signal transduction and interactions and thus represent potential therapeutic targets [50–53]. The hub is defined as a target with a high degree of centrality, while a bottleneck is defined as a target with a high betweenness centrality [52].

Based on the calculated degree and betweenness centrality, biological targets representing hubs and bottlenecks in the network were identified in order of the highest values. Meanwhile, the results of studies on the relationship between screened biological targets and phenotypes were collected from a literature review. This evidence collected from the literature was used, together with the known efficacy of the candidates against the target disease, to infer their potential therapeutic effects. Additionally, identified compound targets were supported by biological pathways and literature review analyses. These results validated the potential herbal combinations targeting a disease by determining the likelihood of the compound targets acting on the disease, thus supporting the herb-level results.

### *In vitro* validation

*In vitro* validation focused on asthma, the phenotype with the highest relative proximity in our predictive analysis. We used RBL-2H3 cells, a rat basophilic leukemia cell line commonly applied in asthma-related studies, cultured in Eagle’s minimum essential medium with 10% fetal bovine serum. Cells were treated with selected asthma-related compounds (d-tryptophan, carvacrol, and limonene) at IC₂₀ (20% inhibitory concentration) levels, determined via MTT (3-[4,5-dimethylthiazol-2-yl]-2,5-diphenyl-2H-tetrazolium bromide) viability assays. Next, β-hexosaminidase release assays were performed following IgE sensitization and antigen stimulation to model asthma-like responses. Gene expression analysis was subsequently conducted using quantitative real-time PCR (qRT-PCR) to measure key biomarkers associated with asthma-related inflammatory reactions. The data presented for the *in vitro* experiments represent the results of three independent replications. Comparisons between groups were conducted using one-way analysis of variance (ANOVA) with normality tests and Duncan’s multiple range tests as high-power *post hoc* tests to measure differences identified via ANOVA, by Statistical Package for the Social Sciences (SPSS 20, SPSS Inc., Chicago, IL) software. Results are expressed as the mean ± the standard error of the mean (SEM), with the statistical significance set at *P* <.05. Primer sequences and detailed experimental conditions are provided in Supplementary Section A.

## Results

### Inferred herbal material combinations for phenotypes

We conducted an herb–phenotype ARM analysis that had been designed for several phenotypes: asthma, diabetes, arthritis, stroke, and inflammation. The number of inferred herbal combinations per phenotype is described in Supplementary Table S4. The support, confidence, and lift evaluation metrics were calculated for the association rules between herbal material combinations and phenotypes (Supplementary Table S5). For example, the association rule “*Morus alba*, *Ephedra sinica*, *Perilla frutescens*, *Pinellia ternata* → asthma” from our study, with a confidence of 0.67, indicates that ~67% of prescriptions containing this combination are known to be effective for asthma. Moreover, with a lift of 17.88, we can conclude that a significant correlation exists between this combination and asthma. The top 10 inferred association rules for each phenotype are provided in Supplementary Tables S6–S10.

### Effect of the inferred herbal material combinations in the human protein interactome network

**Figure 2:**
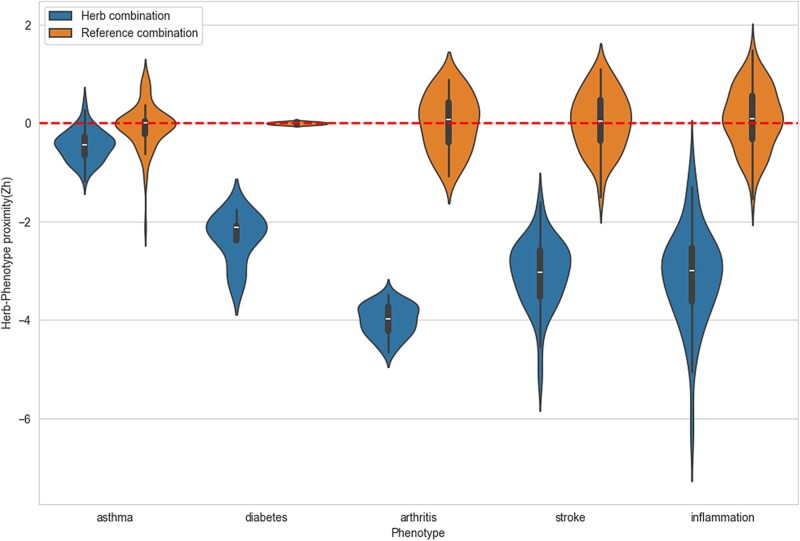
Relative proximity distribution for the top 5% of inferred association rules (i.e. herbal material combinations) and calculated reference distances for each phenotype (asthma, diabetes, arthritis, stroke, and inflammation). Statistical testing using Student’s *t*-test or the Mann–Whitney *U* test revealed significant differences in the means across all five phenotypes.

We calculated the herb–phenotype proximity for the inferred herbal material combinations and identified key proteins that acted as hubs or bottlenecks (Table 1). The results revealed that all herbal materials for each association rule satisfied the predefined proximity threshold (${z}_h\le -0.15$), indicating that each combination was strongly related to the corresponding phenotype in the rule. These results were confirmed by violin plots comparing the mean herb–phenotype proximity for each combination and phenotype to reference combinations (Fig. 2). The reference combination for each herbal material combination was the set of expected distances between two randomly selected groups. In the box plots, the *z*-scores for the herbal material combination for each phenotype averaged −2.59. This was much lower than the average proximity of reference combinations, which was 0.01. Therefore, we tested the hypothesis to validate the statistical differences between the two groups. For the four phenotypes other than diabetes, we employed Student’s *t*-test statistical analysis and observed statistically significant differences in means for all cases (*P* < .0001). However, owing to the limited number of association rules, we used the Mann–Whitney *U* test for diabetes and identified a statistically significant difference in means (*P* = .0019). These findings are consistent with previous studies demonstrating that frequently used herbal material combinations tend to exhibit a shorter network proximity than randomly assembled pairs [54].

**Table 1:** Results of the herb-level analysis for each phenotype

Phenotype	Herbal material	Herb–phenotype proximity (${z}_h$)
Asthma	*Morus alba*	–0.17
	*Ephedra sinica*	–0.43
	*Perilla frutescens*	–0.19
	*Pinellia ternata*	–1.20
Diabetes	*Trichosanthes kirilowii*	–2.59
	*Nelumbo nucifera*	–2.59
	*Dioscorea polystachya*	–1.02
	*Rehmannia glutinosa*	–2.13
Arthritis	*Gentiana macrophylla*	–2.40
	*Angelica pubescens*	–3.92
	*Achyranthes bidentata*	–4.48
Stroke	*Acorus gramineus*	–2.02
	*Pinellia ternata*	–4.34
	*Arisaema amurense*	–1.85
Inflammation	*Manis pentadactyla*	–3.34
	*Citrus unshiu*	–3.38
	*Gleditsia sinensis*	–2.59

Table presenting herb–phenotype proximity scores (${\boldsymbol{z}}_{\boldsymbol{h}}$) for each inferred herbal material across five phenotypes (asthma, diabetes, arthritis, stroke, and inflammation) derived from herb-level analysis.

We also found that the key hubs and bottlenecks mostly correspond with the herb targets proximal to the phenotype in the herb–phenotype network analysis (Supplementary Table S11). For the average of the 5.53 genes identified as hubs or bottlenecks in the structural network analysis, approximately three genes appeared in the list of top 10 herb targets close to the phenotypes in the herb–phenotype network analysis.

Using a literature review, we investigated the mechanisms through which herbal material combinations affect the corresponding phenotypes (Supplementary Table S12). Based on these results, it can be concluded that the identified combinations can likely modulate phenotypes through cumulative interactions with their herb targets.

### Compound-level analysis of the inferred combinations

**Figure 3:**
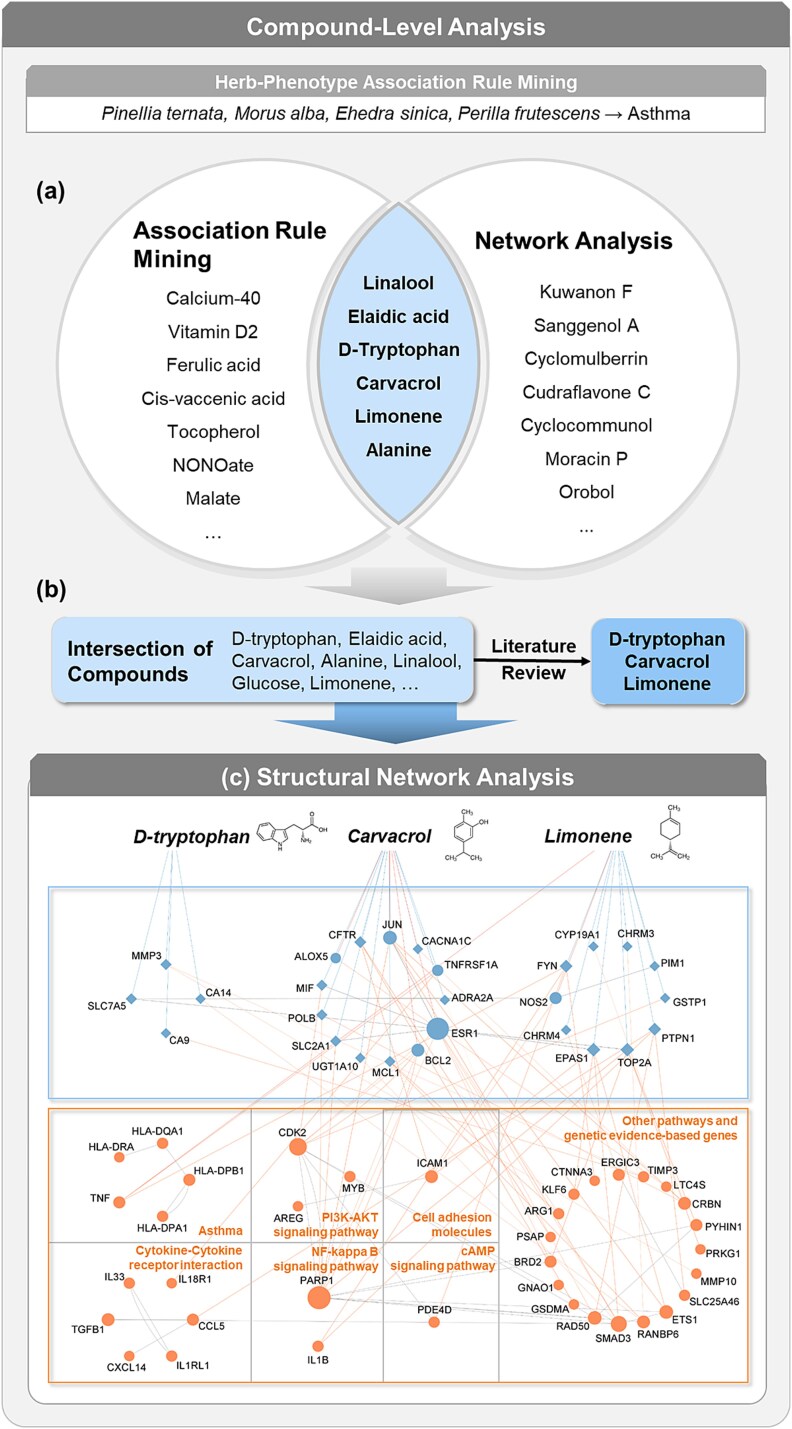
Compound-level analysis results based on inferred herbal material combinations for asthma. (a) Compound–phenotype ARM and compound–phenotype network analyses were performed, and the 16 compounds found in both were extracted. (b) Three of these 16 compounds (d-tryptophan, carvacrol, and limonene), known to be useful in treating asthma, were selected based on a literature review. (c) Structural network analysis categorized nodes into compound targets (top) and phenotype-related genes (bottom). Nodes with compound targets and phenotype-related genes are presented by circular nodes instead of the rhombus shape used for other compound targets. Edges between the top and bottom boxes represent interactions between compound targets and phenotype-related genes, while edges between compound names and circular nodes indicate interactions between compounds and phenotype-related genes. Phenotype-related genes were classified into seven categories based on the biological pathway to which they belong.

We also conducted a compound-level analysis of the inferred herbal material combinations (Fig. 3). The initially inferred number of compounds is described in Supplementary Table S4. The compounds within the intersection between the top 150 association rules, ranked by high confidence values, obtained from the compound–phenotype ARM analysis and the compounds related to herbal material combinations obtained from the compound-level network analysis were identified (Fig. 3a). The top 150 association rules were notable because their average confidence values were higher than the overall average confidence of the generated association rules. A literature review was then conducted on the compounds that were identified in the intersection of these analyses to determine and those that have previously been reported to promote potential pharmacological effects on the target phenotype (Fig. 3b). Next, a structural network analysis of compound targets identified in the literature review was conducted, and the phenotype-related biological pathways and supporting evidence, acquired via the Kyoto Encyclopedia of Genes and Genomes (KEGG) manual curation [55] and the literature review analyses, were assessed (Fig. 3c). We also evaluated the consistency between the herb- and compound-level analyses by determining the overlap ratio for the compounds identified using ARM and those identified using the network analysis (Supplementary Table S13). The presence of the three compounds in the identified herbal material combinations is detailed in Supplementary Table S14. These results revealed that the identified herb targets were associated with multiple pathways related to the phenotype (Table 2, Supplementary Tables S15–S17). This evidence suggests that our method can be used to investigate the molecular mechanisms of compound targets and provides an opportunity to more clearly understand the relationship between compounds and phenotypes based on their MoAs in humans. The compound-level analysis and literature review results for the five phenotypes are presented in Supplementary Section B with Figs. S1–S4.

**Table 2:** Results of structural network analysis and biological pathway searches for asthma

Phenotype	Compounds identified in the literature review	Hub	Bottleneck	Related biological pathways
Asthma	D-tryptophan	SLC7A5, MMP3, CA9	SLC7A5	Other pathways and genetic evidence-based genes
	Carvacrol	ESR1, JUN, BCL2	ESR1	Asthma, PI3K–AKT signaling pathway, NF-kappa B signaling pathway, T cell receptor signaling pathway, other pathways, and genetic evidence-based genes
	Limonene	ESR1, EPAS1, TOP2A	ESR1, PTPN1	Asthma, PI3K–AKT signaling pathway, cell adhesion molecules, cytokine–cytokine receptor interaction, NF-kappa B signaling pathway, other pathways, and genetic evidence-based genes

Table of the structural network analysis and biological pathway mapping results performed for the three compounds inferred for asthma, along with the identified hub and bottleneck proteins.

### *In vitro* verification of the compound-level analysis

**Figure 4:**
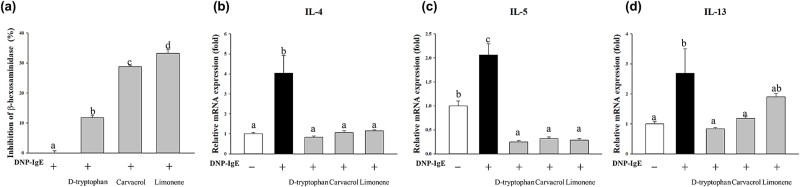
The *in vitro* validation results in the compound-level analysis. (a) Inhibitory effects on β-hexosaminidase. (b–d) mRNA expression levels of IL-4, IL-5, and IL-13—proinflammatory cytokines related to the predicted biological pathways—under d-tryptophan, carvacrol, and limonene treatment. The letters above the bars indicate significant differences (Duncan’s multiple range test; *P* < .05). The value orders from small to large are presented alphabetically.

Since asthma exhibited the highest relative proximity, reflecting the most ambiguous results from our analysis, we specifically selected asthma-related compounds for further *in vitro* validation to strengthen the reliability and robustness of our integrative framework. First, the IC_20_ values for the three compounds identified using the structural network analysis were calculated from MTT assays to establish the optimal concentration for treatment (Fig. S5). The intracellular activity of β-hexosaminidase, a marker enzyme for histamine-containing granules in mast cells, was measured (Fig. 4). As shown in Fig. 4a, d-tryptophan, carvacrol, or limonene treatment significantly reduced β-hexosaminidase levels in IgE–antigen complex-stimulated mast cells by 11.75%, 28.78%, and 33.22%, respectively, compared to vehicle-treated cells (*P* < .001), indicating a suppressive effect on degranulation. The inhibitory effects of the selected compounds on the elevated interleukin (IL-4, IL-5, and IL-13) mRNA levels induced by the IgE–antigen complex were also tested. d-Tryptophan, carvacrol, and limonene significantly inhibited IL-4 mRNA levels compared to the IgE–antigen complex-stimulated group (0.21-, 0.26-, and 0.28-fold, respectively, *P* < .001; Fig. 4b), as were IL-5 mRNA levels (0.12-, 0.15-, and 0.14-fold, respectively, *P* < .001; Fig. 4c). However, while the IL-13 expression was significantly inhibited by d-tryptophan (0.31-fold) and carvacrol (0.43-fold), no notable effect was promoted by limonene (*P* < .05; Fig. 4d). These results highlight the anti-inflammatory potential of the identified compounds.

qRT-PCR was also conducted to identify the hub and bottleneck genes associated with the structural network analysis for asthma (Fig. S6). Subsequently, qRT-PCR found that, compared to the IgE–antigen complex-stimulated group, d-tryptophan, carvacrol, and limonene reduced the mRNA levels of five target proteins identified in the compound-level analysis. Moreover, these changes were observed only for the respective targeted compound, highlighting their specific effects.

## Discussion

Herbal material combinations are used in TM to treat various diseases. However, the formulas for combining ingredients remain poorly organized, and the complex mechanisms involved in integrating multiple compounds in herbal materials make understanding the pharmacological relationships in TM difficult. *In silico* approaches have been proposed to analyze TM and herbal materials; nonetheless, these approaches either require large volumes of high-quality structural and ligand property data or are limited by interpretability challenges and the restricted analytical scope. In particular, these *in silico* approaches primarily focus on identifying effective single active ingredients against disease [56–58].

To overcome these issues, this study proposed an approach that integrated ARM and network pharmacology analyses to identify potential therapeutic herbal material combinations and compounds for various diseases. Based on the synthesis of large volumes of data on compounds, proteins, phenotypes, and their interactions, this approach facilitates the highly interpretable analysis of diseases to understand the polypharmacological properties of TM candidates and their MoAs in humans. Specifically, the network proximity and structural network analyses provide a mechanistic basis for understanding how the candidates interact with a target phenotype, with additional evidence supplied by the literature review.

Our study hypothesized that, if certain herbal material combinations frequently appear in prescriptions for a particular phenotype, these combinations are likely to play an important role in any therapeutic effect on that phenotype. Based on this hypothesis, we found that the proposed framework can effectively screen candidate herbal material combinations with potential therapeutic effects. For example, TM prescriptions containing the identified herbal material combinations for asthma were included in prescriptions that have been experimentally demonstrated to help inhibit the secretion of airway mucus and prevent airway smooth muscle contractions [59, 60]. In addition, the individual herbal materials in this combination have been shown to relieve asthma [61–64]. Thus, employing the proposed framework for screening can reduce the time and effort required to obtain useful results.

The proposed approach also assumed that, if consistent results were observed in the herb- and compound-level analyses of candidate compounds against a disease and could be validated, those compounds and associated herbal materials could be useful for treating the target disease. Subsequently, we demonstrated that ARM and network-based proximity analyses can be used to derive potential therapeutic compounds via intersection analysis. Thus, compared to traditional ligand- and structure-based approaches, these ARM and network-based analyses can serve as the basis for understanding the pharmacological effects of herbal materials that comprise multiple compounds with minimal effort [65, 66]. Since manually determining the compounds or proteins that promote the therapeutic effects against a phenotype when handling a large volume of unclassified TM data remains difficult, our proposed framework can act as the primary means for information collection and utilization during drug development studies. These results also showed that the screened compounds and compound targets interacted with multiple pathways associated with the phenotype, alongside various evidence from past studies. These compound-level analysis results tend to be consistent with the herb-level analysis results, supporting the efficacy of the inferred combinations.

Overall, our proposed framework can be employed to discover effective candidates from TM for use in treating various diseases. By integrating multiple levels of information to assess the potential pharmacological effects of herbal material combinations and compounds, active herbal ingredients can be identified while considering their pharmacokinetic properties [67]. In addition, the compound- and molecular-level-derived evidence of interactions between herbal material combinations and diseases and the potential biological pathways involved can be utilized in other *in silico* studies. Moreover, *in vitro* assays for evaluating human phenotypic efficacy can serve as valuable methods [68]. For example, an IgE–antigen-induced, RBL-2H3, basophilic leukemia cell model was employed in this study to validate the efficacy of the predicted compounds in treating asthma [69, 70]. The results of the *in vitro* analysis confirmed the predictions of the compound-level analysis, which effectively relieved the symptoms of an allergic response in mast cells. The suppressed release of β-hexosaminidase from RBL-2H3 cells, which is typically triggered by IgE–antigen complexes as mast cell activators, highlighted the inhibitory effects of the selected compounds on mast cell degranulation and the release of histamine-containing granules, which is a key inflammatory response associated with asthma [71]. In addition, in chronic asthma, Th2 cells are known to infiltrate the lungs and produce inflammatory cytokines, such as IL-4, IL-5, and IL-13, which are crucial in initiating and progressing allergic diseases, including asthma, by driving Th2-mediated immune responses [72].

While the present study integrates computational and *in vitro* approaches to validate the predicted therapeutic potential of selected candidates and gene expression markers based on network topological features, we acknowledge the limitations of literature-based validation methods. Nevertheless, this proposed analytical method requires further improvement before implementation as an effective prescreening tool in real clinical practice. Most studies, including our proposed approach, do not separately consider parts or the dosage of the material in the assessment. In particular, variations in the ratios between herbal materials are currently difficult to analyze because ARM is suited for categorical variables, and many prescriptions lack detailed compositional data. Furthermore, insufficient data on processing methods and the influence of these methods on pharmacological effects represents another significant limitation that may affect the results of the proposed method. In addition, the curse of dimensionality, which is caused by the large amount of item set vectors for a compound compared to the herbal material, makes identifying potential compound combinations difficult. However, these issues can be improved by accumulating additional knowledge in future studies. Moreover, our method relied heavily on existing findings and lacked direct experimental confirmation, which may have limited the accuracy of the predictive model. Furthermore, the results of these analyses, including the literature review, are limited to evaluating the efficacy of the candidate; thus, there remains a need to incorporate metrics that assess safety in future studies [73]. To address these limitations, we propose further validation studies using *in vivo* models or human exposure to provide clearer mechanistic insights and improve the robustness of the proposed predictive framework. Indeed, suppose clinical trials validate the safety and efficacy of identified candidates, then these trials can be employed in drug combinations, ethnopharmacological diets, TM prescriptions, and/or dietary supplements with multi-target properties.

## Conclusion

The herbal materials used in TM and their compounds offer many potential benefits in the development of modern medicine. Our proposed integrative method utilizes multiple levels of data to conduct a large-scale analysis of potential TM ingredients. Our method can also provide evidence for pharmacological effects through topological properties and the MoAs of the inferred candidates in the human protein interactome network. We believe our process will aid drug discovery in TM and contribute to a better understanding of the polypharmacological characteristics of herbal materials.

**Key Points**

- Multi-level association rule mining provides polypharmacological candidates.
- Applicable to a wide range of diseases through integrated large-scale analysis.
- High interpretability with network pharmacology at the molecular layer.
- Mechanistic evidence enhancing the understanding of polypharmacological effects.

## Supplementary Material

250617_Supplementary_materials_bbaf328

## Data Availability

The code used in this study is available at GitHub (https://github.com/bmil-jnu/InPETM) along with the pre-processed data. The UMLS identifiers of the phenotypes used for the analysis are freely accessible with a UMLS license request from the National Library of Medicine (NLM).
