# Supplementary material for: Multi-level association rule mining and network pharmacology to identify the polypharmacological effects of herbal materials and compounds in traditional medicine
Source: Brief Bioinform. 2025 Jul 7;26(4):bbaf328. doi: 10.1093/bib/bbaf328 (PMC12232419; doi:10.1093/bib/bbaf328)
Supplement: 250617_Supplementary_materials_bbaf328 [file 250617_supplementary_materials_bbaf328.docx]

**Supplementary Material**

Table S1. Number of entities collected from each database.

| **Database** | **Prescriptions** | **Herbal materials** | **Compounds** | **Genes** | **Phenotypes** |
| --- | --- | --- | --- | --- | --- |
| KTKP^a^ | 20,121 | 5500 | 30,695 | Unknown | 12,500 |
| TCM-ID^b^ | 7443 | 2751 | 7,375 | 768 | 366 |
| KampoDB | 298 | 180 | 3002 | 62,906 | 189 |
| COCONUT^c^ | 30,273 | 2,295,525 | 64,966,140 | 20,503,546 | 366,488 |

^a^Korean Traditional Knowledge Portal; ^b^Traditional Chinese Medicine Information Database; ^c^Compound Combination-Oriented Natural Product Database with Unified Terminology

Table S2. Number of relations collected from each database.

| **Database** | **Prescription**–**herb** | **Prescription**–**phenotype** | **Herb**–**compound** | **Herb**–**phenotype** | **Compound**–**phenotype** | **Compound**–**gene** | **Gene**–**phenotype** |
| --- | --- | --- | --- | --- | --- | --- | --- |
| KTKP^a^ | 127,672 | 66,664 | 41,672 | 38,580 | - | - | - |
| TCM-ID^b^ | 5615 | 4792 | 1357 | 3941 | - | - | - |
| KampoDB | 469 | 944 | - | - | - | - | - |
| COCONUT^c^ | 136,710 | 77,877 | 2,003,112 | 568,916 | 5,805,762 | 85,556,744 | 3,396,958 |
| DrugBank | - | - | - | - | 5590 | 38,132 | - |
| TTD^d^ | - | - | - | - | 7123 | - | - |
| CTD^e^ | - | - | - | - | 5,752,348 | 1,965,902 | 41,314 |
| BindingDB | - | - | - | - | - | 161,145 | - |
| STITCH^f^ | - | - | - | - | - | 14,147,898 | - |
| FooDB | - | - | 1,415,847 | - | - | 94,834 | - |
| CMAUP^g^ | - | - | 221,323 | - | - | 13,982 | - |

^a^Korean Traditional Knowledge Portal; ^b^Traditional Chinese Medicine Information Database; ^c^Compound Combination-Oriented Natural Product Database with Unified Terminology; ^d^Therapeutic Target Database; ^e^Comparative Toxicogenomics Database; ^f^Search Tool for Interacting Chemicals; ^g^Collective Molecular Activities of Useful Plants

We mapped and standardized the collected data according to the international identifiers used by each database, where possible. However, since no unified, internationally used identifiers existed, these data were integrated for prescriptions based on the collected names. Importantly, we recognized that prescriptions with the same name may vary in composition; thus, we treated prescriptions with identical names but differing herbal materials as distinct entries. Meanwhile, metal and mineral sources were removed for herbal materials due to the lack of international identifiers and nomenclature uniformity across databases; moreover, taxonomy data from the National Center for Biotechnology Information (NCBI) were used for their biological classification [1]. We acquired NCBI classification data at the species and infraspecific (i.e., subspecies, variety, or forma) levels and then unified the infraspecific classifications into the species level. Compounds were mapped to PubChem [2] or ChEMBL [3] identifiers, while genes were mapped using species-specific NCBI Entrez Gene identifiers [4]. The collected phenotype data were standardized according to the Unified Medical Language System (UMLS), which summarizes biomedical terminology in a hierarchical structure, based on name–entity recognition using MetaMap [5, 6].

To effectively extract prescriptions related to specific phenotypes, those phenotypes at similar or lower-levels in the UMLS hierarchical structure were integrated with higher-level phenotypic data. For example, we integrated rheumatoid arthritis and osteoarthritis, which have relatively low hierarchical levels, into arthritis, which has a higher hierarchical level. This broadened the categories for individual phenotypes and allowed the relationships between phenotypes to be more easily investigated [7].

**A. Detailed Experimental Conditions of *In* *Vitro* Validation**

**1. Materials and Reagents**

Dulbecco's phosphate-buffered saline (DPBS), fetal bovine serum (FBS), and HEPES buffer solution were purchased from Biowest (Nuaillé, Cholet, France). Penicillin–streptomycin (PS) was obtained from Gibco (Rockville, MD, USA). Dimethyl sulfoxide (DMSO), bovine serum albumin (BSA), and 2,5-diphenyltetrazolium bromide (MTT) were purchased from Sigma‒Aldrich (St. Louis, MO, USA). D-tryptophan, carvacrol, and limonene were also obtained from Sigma‒Aldrich (USA). All tested compounds were of HPLC grade or primary reference standards.

**2. Cell Cultures**

RBL-2H3, basophilic leukemia cells from *Rattus norvegicus*, were obtained from ATCC (Manassas, VA, USA) and maintained in ATCC-formulated Eagle's Minimum Essential Medium (Catalog No. 30-2003, ATCC) with 10% FBS and 100 U/mL PS. Cells were incubated at 37 °C in a humidified atmosphere with 5% CO₂. For the experiments, RBL-2H3 cells were seeded into 96-well plates or 12-well plates (SPL, Gyeonggi, Korea), as required. Cell cultures were used between passages 19 and 24, with the medium changed every 2 to 3 days. All experiments were conducted in triplicate for statistical analysis.

**3. Cell Viability Measurements**

RBL-2H3 cells were seeded into 96-well plates at a density of 1 × 10⁵ cells/mL and incubated for 24 h. The cells were then treated with the test compounds at concentrations ranging from 6.25 μM to 100 μM, prepared through stock solution dilutions in the culture medium. Control wells received an equivalent volume of the culture medium. After 24 h of treatment, the medium was removed, and 20 μL of MTT solution (5 mg/mL in phosphate-buffered saline (PBS)) was added to each well. The cells were then incubated at 37 °C for 4 h. After 100 μL of DMSO was added, the optical density (OD) was measured at 570 nm using a microplate reader. IC₂₀ values, which represent the concentrations required for 20% viability inhibition, were calculated using a sigmoidal, logistic, four-parameter equation based on the logarithmic concentration and relative viability compared to the control.

**4. β-Hexosaminidase Inhibition Assays**

RBL-2H3 cells (2 × 10^5^) were sensitized in 0.05 μg/mL DNP–IgE (Sigma), treated with IC₂₀ concentrations of D-tryptophan, carvacrol, or limonene for each group, and incubated for 24 h. After washing with Ca^2+^ and Mg^2+^ in DPBS buffer, the cells were incubated with 0.1% BSA. Subsequently, 0.05 μg/mL DNP–BSA (Sigma) was added, and the cells were incubated for 2 h, after which the cell lysates were collected. Next, 25 μL of 5 mM 4-nitrophenyl N-acetyl-β-D-glucosaminide solution was added to each sample, and the samples were incubated for 110 min. After incubation, 200 μL of 0.05 M sodium carbonate buffer was added to halt the reaction, and the OD was measured at 405 nm. The percentage of β-hexosaminidase inhibition was calculated using Equation (S1):

$$\begin{aligned} \mathrm{Inhibition}\left( \% \right)= \left( 1- \frac{\mathrm{OD}\left( T \right)-OD (N)}{\mathrm{OD}\left( C \right)-OD (N)} \right)\times100\#\left( S1 \right) \end{aligned}$$

where *N* is (IgE(-) + DNP-HSA(-) + sample(-)), *C* is (IgE(+) + DNP–HSA(+) + sample(-)), and *T* is (IgE(+) + DNP–HSA(+) + sample(+)).

**5. mRNA Extraction and Quantitative Real-Time PCR**

Total mRNA was extracted from the treated cells using TRIzol reagent (Life Technologies, Rockville, MD, USA) according to the manufacturer’s protocol. The extracted mRNA was diluted to a 400–800 ng/mL concentration using nuclease-free water. Complementary DNA (cDNA) was synthesized immediately using a High-Capacity RNA-to-cDNA Synthesis kit (Hoffmann-La Roche Ltd., Basel, Switzerland). Quantitative real-time PCR (qRT-PCR) was conducted using a StepOnePlus™ Real-Time PCR System (Hoffmann-La Roche Ltd.). The comparative 2^−ΔΔCT^ method was employed to quantify the relative mRNA expression levels normalized against GAPDH as the internal control. The primer sequences for the target genes are presented in Supplementary Table S3.

Table S3. Primer sequences used for qRT-PCR (*Rattus norvegicus*).

| **Gene** |  | **Primer sequence (5’–3’)** |
| --- | --- | --- |
| GAPDH | Forward | TGTGAACGGATTTGGCCGTA |
|  | Reverse | GATGGTGATGGGTTTCCCGT |
| IL-4 | Forward | GTACCGGGAACGGTATCCAC |
|  | Reverse | GTGAGTTCAGACCGCTGACA |
| IL-5 | Forward | ACGATGAGGCTTCCTGTTCC |
|  | Reverse | CTGGTCTTCCGCCTCTCTTC |
| IL-13 | Forward | AACCAAAAGGCCTCGGATGT |
|  | Reverse | GGCCATAGCGGAAAAGTTGC |
| SLCA5 | Forward | GTGGGGAACATTGTGTTGGC |
|  | Reverse | ACCTGCAGGATCCATTTGGG |
| MMP3 | Forward | TTTGGCCGTCTCTTCCATCC |
|  | Reverse | GCATCGATCTTCTGGACGGT |
| CA9 | Forward | CCGTAACCACGTAACCACGA |
|  | Reverse | CCCAGCACTAAGAAGGCACA |
| ESR1 | Forward | CTGGTGCAACAAGGCCATTC |
|  | Reverse | AAGCCAATCTGTACCTCGGC |
| JUN | Forward | CCAACCAACGTGAGTGCAAG |
|  | Reverse | GAGGGCATCGTCGTAGAAGG |
| BCL2 | Forward | AGCATGCGACCTCTGTTTGA |
|  | Reverse | TCACTTGTGGCCCAGGTATG |
| EPAS1 | Forward | GCTCTCTTTTGGCGTCTTGC |
|  | Reverse | TGTGTTCGCAGGAAGCTGAT |
| TOP2A | Forward | CTCCTGTGGGGAAAAGGGTC |
|  | Reverse | GTGGAGCTCTTCTGTGGCTT |
| PTPN1 | Forward | CACAGTACGGCAGTTGGAGT |
|  | Reverse | ATTGAGGGGGCTCTGCTTTC |

Table S4. Number of inferred herbal material combinations and compounds from the herb- and compound-level analyses.

| **Phenotype** | **Herbal material combinations^a^** | **Compounds** |
| --- | --- | --- |
| Asthma | 1623 | 1833 |
| Diabetes | 199 | 1916 |
| Arthritis | 733 | 1797 |
| Stroke | 1373 | 2656 |
| Inflammation | 1539 | 1977 |

^a^ indicates the number of inferred herbal material combinations, not individual herbal materials. Each combination comprised two to four herbal materials.

Table S5. Inferred therapeutic combinations of herbal materials for each phenotype.

| **Association rule** | **Support** | **Confidence** | **Lift** |
| --- | --- | --- | --- |
| *Morus alba, Ephedra sinica, Perilla frutescens, Pinellia ternata* → asthma | 0.0006 | 0.67 | 17.88 |
| *Trichosanthes kirilowi, Nelumbo nucifera, Dioscorea polystachya, Rehmannia glutinosa* → diabetes | 0.0006 | 1.00 | 202.84 |
| *Gentiana macrophylla, Angelica pubescens, Achyranthes bidentata* → arthritis | 0.0006 | 0.43 | 39.94 |
| *Acorus gramineus, Pinellia ternata, Arisaema amurense* → stroke | 0.0009 | 0.64 | 22.46 |
| *Manis pentadactyla, Citrus unshiu, Gleditsia sinensis* → inflammation | 0.0008 | 0.73 | 16.12 |

Table S6. Top 10 inferred association rules for asthma and the related prescriptions.

| **Association rule** | **Support** | **Confidence** | **Lift** | **Prescriptions containing the inferred association rule** |
| --- | --- | --- | --- | --- |
| *Morus alba, Ehedra sinica, Perilla frutescens, Pinellia ternata* → asthma | 0.0006 | 0.6667 | 17.8827 | Haengsoeum, Bunsingieum, Maduryeongsan, Bungieum, Bungijasoeum, Insamgwanhwago |
| *Platycodon grandiflorus, Morus alba, Perilla frutescens, Areca catechu* → asthma | 0.0006 | 0.6667 | 17.8827 | Haecheonsingitang, Sangseogyeongheombang, Chihyosan, Haepyoijintang, Cheongeumjeongcheontang, Pyeongpyetang, Jasoeumja, Jeongcheontang |
| *Platycodon grandiflorus, Morus alba, Ephedra sinica, Glycyrrhiza uralensis* → asthma | 0.0006 | 0.6000 | 16.0944 | Haengsoeum, Sangseogyeongheombang, Hwagaesan, Gamiseunggalpaedoksan, Chihyosan, Maengmundongtang, Haepyoijintang, Gilgyeongsan, Pyeongpyetang |
| *Morus alba, Fritillaria thunbergii, Glycyrrhiza uralensis, Aster tataricus* → asthma | 0.0006 | 0.6000 | 16.0944 | Gamisocheongnyongtang, Sayuktang, Sangseogyeongheombang, Gamiinchamsiwansan, Chihyosan, Haepyoijintang, Okwasan, Jawanyongtang, Maengmundongtang |
| *Pinellia ternata, Morus alba, Ehedra sinica, Glycyrrhiza uralensis* → asthma | 0.0010 | 0.5882 | 15.7788 | Haecheonsingitang, Sangseogyeongheombang, Tupungjinhwacheonggitang, Gamiseunggalpaedoksan, Insamjeongcheontang, Chihyosan, Maengmundongtang, Haepyoijintang, Cheongeumjeongcheontang, Pyeongpyetang |
| *Morus alba, Aster tataricus, Prunus armeniaca, Citrus unshiu* → asthma | 0.0007 | 0.5882 | 15.6473 | Gamisocheongnyongtang, Haengsoeum, Jasobanhatang, Sayuktang, Tupungjinhwacheonggitang, Gamiinchamsiwansan, Chihyosan, Haepyoijintang, Yunpyejesueum, Pyeongpyesan |
| *Platycodon grandiflorus, Morus alba, Ephedra sinica* → asthma | 0.0007 | 0.5833 | 15.6473 | Haengsoeum, Mahwangjeongcheontang, Sangseogyeongheombang, Hwagaesan, Gamiseunggalpaedoksan, Yunpyejesueum, Chihyosan, Maengmundongtang, Haepyoijintang, Gilgyeongsan |
| *Morus alba, Pinellia ternata, Prunus armeniaca, Aster tataricus* → asthma | 0.000895 | 0.5625 | 15.0885 | Gamisocheongnyongtang, Jasobanhatang, Sayuktang, Sangseogyeongheombang, Tupungjinhwacheonggitang, Gamiinchamsiwansan, Chihyosan, Haepyoijintang, Yunpyejesueum, Okwasan |
| *Morus alba, Pinellia ternata, Ephedra sinica* → asthma | 0.0010 | 0.5556 | 14.9022 | Haecheonsingitang, Jeongcheontang, Sangseogyeongheombang, Tupungjinhwacheonggitang, Gamiseunggalpaedoksan, Yunpyejesueum, Insamjeongcheontang, Chihyosan, Maengmundongtang, Haepyoijintang |
| *Wolfiporia cocos, Fritillaria thunbergii, Perilla frutescens, Prunus armeniaca* → asthma | 0.0006 | 0.5455 | 14.6313 | Haecheonsingitang, Gamigeumsuyukgunjeon, Haengsocheonggisan, Gamijihwangtang, Gamiinchamsiwansan, Chihyosan, Sahwacheongpyetang, Cheonggeumtang, Haepyoijintang, Cheongpyeeum |

Table S7. Top 10 inferred association rules for diabetes and the related prescriptions.

| **Association rule** | **Support** | **Confidence** | **Lift** | **Prescriptions containing the inferred association rule** |
| --- | --- | --- | --- | --- |
| *Trichosanthes kirilowii, Nelumbo nucifera, Dioscorea polystachya, Rehmannia glutinosa* → diabetes | 0.0006 | 1.0000 | 202.837 | Gamijihwangtang, Dangnyobyeonggyeongheombang, Gamiseungmabaekchultang, Hyeoldangjeonghwan, Jineumyeonjatang |
| *Dioscorea polystachya, Panax ginseng, Liriope muscari, Trichosanthes kirilowii* → diabetes | 0.0006 | 1.0000 | 202.837 | Dangnyohwan, Gamijihwangtang, Dangnyobyeonggyeongheombang, Gamiseungmabaekchultang, Sunhwangyejilhwangyeongheombang, Jineumyeonjatang |
| *Nelumbo nucifera, Cornus officinalis, Trichosanthes kirilowii* → diabetes | 0.0006 | 1.0000 | 202.837 | Gamijihwangtang, Dangnyobyeonggyeongheombang, Galgeundanggwitang, Gamiseungmabaekchultang, Hyeoldangjeonghwan |
| *Nelumbo nucifera, Rehmannia glutinosa, Cornus officinalis, Trichosanthes kirilowii* → diabetes | 0.0006 | 1.0000 | 202.837 | Gamijihwangtang, Dangnyobyeonggyeongheombang, Galgeundanggwitang, Gamiseungmabaekchultang, Hyeoldangjeonghwan |
| *Dioscorea polystachya, Panax ginseng, Trichosanthes kirilowii* → diabetes | 0.0006 | 1.0000 | 202.837 | Dangnyohwan, Gamijihwangtang, Dangnyobyeonggyeongheombang, Gamiseungmabaekchultang, Sunhwangyejilhwangyeongheombang, Jineumyeonjatang |
| *Nelumbo nucifera, Wolfiporia cocos, Dioscorea polystachya, Trichosanthes kirilowii* → diabetes | 0.0006 | 1.0000 | 202.837 | Dangnyohwan, Gamijihwangtang, Dangnyobyeonggyeongheombang, Gamiseungmabaekchultang, Hyeoldangjeonghwan |
| *Nelumbo nucifera, Dioscorea polystachya, Trichosanthes kirilowii* → diabetes | 0.0007 | 0.8750 | 177.482 | Dangnyohwan, Gamijihwangtang, Dangnyobyeonggyeongheombang, Gamicheongsimyeonjaeum, Gamiseungmabaekchultang, Hyeoldangjeonghwan, Jineumyeonjatang |
| *Nelumbo nucifera, Wolfiporia cocos, Trichosanthes kirilowii* → diabetes | 0.0006 | 0.8571 | 173.860 | Dangnyohwan, Gamijihwangtang, Dangnyobyeonggyeongheombang, Gamiseungmabaekchultang, Hyeoldangjeonghwan, Bojunghwadamjeon |
| *Dioscorea polystachya, Angelica gigas, Trichosanthes kirilowii*  → diabetes | 0.0006 | 0.8571 | 173.860 | Dangnyohwan, Gamijihwangtang, Dangnyobyeonggyeongheombang, Gamiseungmabaekchultang, Hyeoldangjeonghwan, Rokduju, Jineumyeonjatang |
| *Nelumbo nucifera, Dioscorea polystachya, Liriope muscari, Trichosanthes kirilowii*  → diabetes | 0.0006 | 0.8571 | 173.860 | Dangnyohwan, Gamijihwangtang, Dangnyobyeonggyeongheombang, Gamicheongsimyeonjaeum, Gamiseungmabaekchultang, Hyeoldangjeonghwan, Jineumyeonjatang |

Table S8. Top 10 inferred association rules for arthritis and the related prescriptions.

| **Association rule** | **Support** | **Confidence** | **Lift** | **Prescriptions containing the inferred association rule** |
| --- | --- | --- | --- | --- |
| *Gentiana macrophylla, Angelica pubescens, Achyranthes bidentata* → arthritis | 0.0006 | 0.4286 | 39.9372 | Yupungyangyeongtang, Samojingyotang, Haenggimangeumtang, Hwangoldan, Dokhwalgisaengtang, Sambitang, Sopungeum, Mangeumtang, Samojingutang, Gamidaeganghwaltang |
| *Gentiana macrophylla, Angelica pubescens, Angelica gigas, Achyranthes bidentata* → arthritis | 0.0006 | 0.4286 | 39.9372 | Yupungyangyeongtang, Samojingyotang, Haenggimangeumtang, Hwangoldan, Dokhwalgisaengtang, Sambitang, Sopungeum, Mangeumtang, Samojingutang, Gamidaeganghwaltang |
| *Angelica pubescens, Panax ginseng, Achyranthes bidentata, Eucommia ulmoides* → arthritis | 0.0006 | 0.4000 | 37.2748 | Samojingyotang, Hogolju, Dokhwalgisaengtang, Sambitang, Gamisipjeondaebotang, Gamiogapijihwangtang, Gamidaebotang, Mangeumtang, Dokwaltang, Samojingutang, Geonbohojamhwan |
| *Ligusticum chuanxiong, Saposhnikovia divaricata, Angelica pubescens, Achyranthes bidentata* → arthritis | 0.0006 | 0.3750 | 34.9451 | Yupungyangyeongtang, Hogolju, Haenggimangeumtang, Dokhwalgisaengtang, Sambitang, Gamisipjeondaebotang, Sopungeum, Yupungjoseupwadamhwan, Gamidaebotang, Mangeumtang, Isamipaedoksan, Isipsamigyeonggakwan |
| *Angelica pubescens, Angelica gigas, Panax ginseng, Achyranthes bidentata* → arthritis | 0.0006 | 0.3750 | 34.9451 | Samojingyotang, Hogolju, Dokhwalgisaengtang, Sambitang, Gamisipjeondaebotang, Gamiogapijihwangtang, Gamidaebotang, Mangeumtang, Dokwaltang, Samojingutang, Geonbohojamhwan |
| *Panax ginseng, Angelica pubescens, Achyranthes bidentata* → arthritis | 0.0006 | 0.3750 | 34.9451 | Samojingyotang, Hogolju, Dokhwalgisaengtang, Sambitang, Gamisipjeondaebotang, Gamiogapijihwangtang, Gamidaebotang, Mangeumtang, Dokwaltang, Samojingutang, Geonbohojamhwan |
| *Gentiana macrophylla, Panax ginseng, Glycyrrhiza uralensis, Eucommia ulmoides* → arthritis | 0.0006 | 0.3529 | 32.8895 | Yupungtang, Samojingyotang, Dokhwalgisaengtang, Sambitang, Mangeumtang, Samojingutang, Ganghwaryupungtang |
| *Angelica pubescens, Asarum sieboldii, Panax ginseng, Eucommia ulmoides* → arthritis | 0.0006 | 0.3529 | 32.8895 | Yupungtang, Dokhwalgisaengtang, Sambitang, Mangeumtang, Ganghwaryupungtang |
| *Gentiana macrophylla, Angelica pubescens, Panax ginseng, Eucommia ulmoides* → arthritis | 0.0006 | 0.3529 | 32.8895 | Yupungtang, Samojingyotang, Dokhwalgisaengtang, Sambitang, Mangeumtang, Samojingutang, Ganghwaryupungtang |
| *Gentiana macrophylla, Rehmannia glutinosa, Angelica pubescens, Eucommia ulmoides* → arthritis | 0.0006 | 0.3529 | 32.8895 | Yupungtang, Samojingyotang, Dokhwalgisaengtang, Sambitang, Mangeumtang, Samojingutang, Ganghwaryupungtang |

Table S9. Top 10 inferred association rules for stroke and the related prescriptions.

| **Association rule** | **Support** | **Confidence** | **Lift** | **Prescriptions containing the inferred association rule** |
| --- | --- | --- | --- | --- |
| *Acorus gramineus, Pinellia ternata, Arisaema amurense* → stroke | 0.0009 | 0.6429 | 22.4642 | Chimhyanghwagidan, Jeongganhwan, Yeongjihwadamtang, Cheongsindodamtang, Tonggisangyeonhwan, Cheokdamtang, Seopsaengtang, Cheongyeolhwadamtang, Yangyeongtang, Seopsaengeum |
| *Panax ginseng, Ephedra sinica, Prunus armeniaca, Sinomenium acutum* → stroke | 0.0010 | 0.6250 | 21.8402 | Bujasongmyeongtang, Mahwangsongmyeongtang, Palbohoechuntang, Gyojetang, Gagamsongmyeongtang, Chigakjongbang, Gagamsosongmyeongtang, Manbohoechuntang, Ganghwaryeongyosongmyeongtang, Gyebusongmyeongtang |
| *Ephedra sinica, Cinnamomum aromaticum, Panax ginseng, Sinomenium acutum* → stroke | 0.0010 | 0.5882 | 20.5554 | Bujasongmyeongtang, Mahwangsongmyeongtang, Palbohoechuntang, Gyojetang, Gagamsongmyeongtang, Gagamsosongmyeongtang, Ganghwaryeongyosongmyeongtang, Manbohoechuntang, Gyebusongmyeongtang |
| *Scutellaria baicalensis, Ephedra sinica, Prunus armeniaca, Sinomenium acutum* → stroke | 0.0010 | 0.5882 | 20.5554 | Bujasongmyeongtang, Mahwangsongmyeongtang, Palbohoechuntang, Gyojetang, Gagamsongmyeongtang, Chigakjongbang, Gagamsosongmyeongtang, Manbohoechuntang, Ganghwaryeongyosongmyeongtang, Gyebusongmyeongtang |
| *Scutellaria baicalensis, Aconitum carmichaelii, Ephedra sinica, Glycyrrhiza uralensis* → stroke | 0.0009 | 0.5625 | 19.6561 | Bujasongmyeongtang, Mahwangsongmyeongtang, Palbohoechuntang, Gyojetang, Gagamsongmyeongtang, Chigakjongbang, Gagamsosongmyeongtang, Manbohoechuntang, Ganghwaryeongyosongmyeongtang, Gyebusongmyeongtang |
| *Scutellaria baicalensis, Aconitum carmichaelii, Ephedra sinica* → stroke | 0.0009 | 0.5625 | 19.6561 | Bujasongmyeongtang, Mahwangsongmyeongtang, Palbohoechuntang, Gyojetang, Gagamsongmyeongtang, Chigakjongbang, Gagamsosongmyeongtang, Manbohoechuntang, Ganghwaryeongyosongmyeongtang, Gyebusongmyeongtang |
| *Scutellaria baicalensis, Saposhnikovia divaricata, Aconitum carmichaelii, Ephedra sinica* → stroke | 0.0009 | 0.5625 | 19.6561 | Bujasongmyeongtang, Mahwangsongmyeongtang, Palbohoechuntang, Gyojetang, Gagamsongmyeongtang, Chigakjongbang, Gagamsosongmyeongtang, Manbohoechuntang, Galgeunsongmyeongtang, Daehwallakdan |
| *Ephedra sinica, Cinnamomum aromaticum, Prunus armeniaca, Sinomenium acutum* → stroke | 0.0009 | 0.5625 | 19.6561 | Bujasongmyeongtang, Mahwangsongmyeongtang, Palbohoechuntang, Gyojetang, Gagamsongmyeongtang, Gagamsosongmyeongtang, Manbohoechuntang, Galgeunsongmyeongtang, Ryeonsimsan, Bangpungsan |
| *Scutellaria baicalensis, Aconitum carmichaelii, Ephedra sinica, Panax ginseng* → stroke | 0.0009 | 0.5625 | 19.6561 | Bujasongmyeongtang, Mahwangsongmyeongtang, Palbohoechuntang, Gyojetang, Gagamsongmyeongtang, Chigakjongbang, Gyebusongmyeongtang, Gyejisongmyeongtang, Sosongmyeongtang, Jasuhaeeodan |
| *Saposhnikovia divaricata, Ephedra sinica, Prunus armeniaca, Sinomenium acutum* → stroke | 0.0010 | 0.5556 | 19.4135 | Gagamsosongmyeongtang, Manbohoechuntang, Gyejisongmyeongtang, Yeonsimsan, Sosongmyeongtang, Jasuhaeeodan, Baekosongmyeongtang, Galgeunsongmyeongtang, Ryeonsimsan, Bangpungsan |

Table S10. Top 10 inferred association rules for inflammation and the related prescriptions.

| **Association rule** | **Support** | **Confidence** | **Lift** | **Prescriptions containing the inferred association rule** |
| --- | --- | --- | --- | --- |
| *Manis pentadactyla, Citrus unshiu, Gleditsia sinensis* → inflammation | 0.0008 | 0.7273 | 16.1231 | Jininhwalmyeongeum, Seonbanghwalmyeongeum, Bibangtalmyeongsan, Naesookseoltang, Saenghwaltang, Tangnitunongtang, Maneunghwan, Tangnisodogeum, Tangnisodoksan, Naetakwanggisan |
| *Ostericum grosseserratum, Forsythia suspensa, Astragalus membranaceus, Phellodendron amurense* → inflammation | 0.0008 | 0.5000 | 11.0846 | Gugohwagyeontang, Seungyangigwisan, Gugoseungnyeongdanbang, Naetakwanggitang, Hwangnyeonsodoksan, Naetakganghwaltang, Tangnisan, Honongjangyukgo, Hwangyeonsodogeum, Hwangnyeonsodogeum |
| *Taxillus chinensis, Angelica pubescens* → inflammation | 0.0008 | 0.5000 | 11.0846 | Jintongsan, Ohyangnyeongyotang, Ohyangyeongyotang, Dokhwalgisaengtang, Boksinwon, Sinseonpungyakju, Gamiogapijihwangtang |
| *Manis pentadactyla, Citrus unshiu* → inflammation | 0.0011 | 0.4783 | 10.6027 | Seonbanghwalmyeongeum, Bibangtalmyeongsan, Tongyutang, Naesookseoltang, Singongnaetaksan, Tangnisodogeum, Bogwontongseongsan, Bogwontonggisan, Tangnisodoksan, Geotongsan |
| *Ostericum grosseserratum, Forsythia suspensa, Astragalus membranaceus, Saposhnikovia divaricata* → inflammation | 0.0008 | 0.4706 | 10.4326 | Gugohwagyeontang, Seungyangigwisan, Gugoseungnyeongdanbang, Biyeonbiyeomgyeongheombang, Hwangnyeonsodoksan, Ganghwalgugotang, Naetakganghwaltang, Tangnisan, Honongjangyukgo, Gwigyutang |
| *Forsythia suspensa, Ostericum grosseserratum, Astragalus membranaceus* → inflammation | 0.0010 | 0.4545 | 10.0769 | Gugohwagyeontang, Seungyangigwisan, Gugoseungnyeongdanbang, Sodokwagyeontang, Biyeonbiyeomgyeongheombang, Naetakwanggitang, Hwangnyeonsodoksan, Ganghwalgugotang, Naetakganghwaltang, Tangnisan |
| *Manis pentadactyla, Trichosanthes kirilowii, Gleditsia sinensis* → inflammation | 0.0009 | 0.4500 | 9.9762 | Jininhwalmyeongeum, Iljeonsan, Sinisan, Iljijeon, Sinsuwisaengtang, Hwajeongnaesosan, Hwanhonsan, Saenghwaltang, Cheongeumnaesosan, Gamisodogeum |
| *Angelica dahurica, Manis pentadactyla, Gleditsia sinensis* → inflammation | 0.0008 | 0.4444 | 9.8530 | Jininhwalmyeongeum, Seonbanghwalmyeongeum, Bibangtalmyeongsan, Naesookseoltang, Gamijipaesan, Yangmaeiljesan, Saenghwaltang, Naesosan, Iljeonsan, Tangnitunongtang |
| *Angelica dahurica, Manis pentadactyla, Gleditsia sinensis* → inflammation | 0.0008 | 0.4444 | 9.8530 | Jininhwalmyeongeum, Seonbanghwalmyeongeum, Bibangtalmyeongsan, Naesookseoltang, Gamijipaesan, Hwanhonsan, Saenghwaltang, Sinisan, Iljijeon, Sinsuwisaengtang |
| *Manis pentadactyla, Lonicera japonica, Trichosanthes kirilowii* → inflammation | 0.0009 | 0.4286 | 9.5011 | Seonbanghwalmyeongeum, Bibangtalmyeongsan, Naesookseoltang, Chudoksan, Tangnisodogeum, Cheonhwasan, Gagamhyeorinhwalmyeongeum, Gamijipaetang, Gamijipaesan |

Table S11. Results of the herb-level analysis for each phenotype and the known targets.

| **Phenotype** | **Herbal material** | **Herb targets proximal to the phenotype** | **Hub** | **Bottleneck** | **Herb–phenotype proximity (z*ₕ*)** |
| --- | --- | --- | --- | --- | --- |
| Asthma | *Morus alba* | ESR1, SMAD3, CDK2, BCL2, MMP9, TRPA1, PDE4D, NOS2, PARP1, EGFR | ESR1, PARP1, CDK2 | ESR1, PARP1, CDK2 | -0.17 |
|  | *Ephedra sinica* | CDK2, BCL2, ESR1, PARP1, MMP9, HSD11B2, GSK3B, ELAVL1, APP, AR | ESR1, PARP1, CDK2 | ESR1, PARP1, CDK2 | -0.43 |
|  | *Perilla frutescens* | CDK2, BCL2, ESR1, PARP1, NOS2, MMP9, APP, STAT3, RELA, EGFR | ESR1, PARP1, CDK2 | ESR1, PARP1, CDK2 | -0.19 |
|  | *Pinellia ternata* | ESR1, PARP1, MMP9, APP, EGFR, AR, CDK1, HSP90AB1, GAPDH, PPARG | ESR1, PARP1, APP | ESR1, PARP1 | -1.20 |
| Diabetes | *Trichosanthes kirilowii* | PTGS2, GLO1, PTPN12, PPARG, RELA, PTPN2, FASN, GSK3B, TOP2A, DOT1L | EGFR, RELA, ESR2 | RELA, PPARG, PTGS2 | -2.59 |
|  | *Nelumbo nucifera* | SLC22A6, FTO, PTPN12, PPARG, PIK3CD, PRKCB, PTPN2, RELA, BCL2, APP | MYC, EGFR, RELA | RELA, PPARG, NFKB1 | -2.59 |
|  | *Dioscorea polystachya* | PPARG, PTPN11, PTGS2, TOP1, CA9, CA14, DPP4, TERT, PPARD, PTPN14 | PPARG, PTPN11, TOP1 | PPARG, PTPN11, PTGS2 | -1.02 |
|  | *Rehmannia glutinosa* | SLC22A6, NR112, FTO, PPARG, SRR, PRKCB, APP, ESR1, HSP90AB1, TERT | EGFR, ESR2, ESR1 | PPARG, PRKCB, NR1I2 | -2.13 |
| Arthritis | *Gentiana macrophylla* | PTPN2, STAT3, PTGS1, PTGS2, TOP2A, DPP4, PTPN1, PTPN11, PTPN6, RELA | STAT3, RELA, PTPN2 | STAT3, PTPN2, PTGS2 | -2.40 |
|  | *Angelica pubescens* | TGM2, PTGS2, MIF, ABCB1, PTGS1, GAPDH, PTK2, SMAD3, STAT3, IGF1R | EGFR, APP, HSPA8 | IGF1R, PTK2, SMAD3 | -3.92 |
|  | *Achyranthes bidentata* | PTGS2, PPARG, STAT3, PTPN2, CTSD, CTSL, TLR2, PTGS1, HSD11B1, PTPN11 | STAT3, RELA, PPARG | STAT3, PPARG, PTPN2 | -4.48 |
| Stroke | *Acorus gramineus* | BCL2L1, ALOX5, ESR1, ESR2, FASN, CTSB, CSNK2A1, CSNK2A2, ALPL, MDH1 | BCL2L1, ESR2, MCL1 | BCL2L1 | -2.02 |
|  | *Pinellia ternata* | ADORA1, GPR17, MMP2, ALOX5, MMP9, CDK1, CDK2, ESR1, APP, PARP1 | APP, EGFR, ESR2 | MMP9 | -4.34 |
|  | *Arisaema amurense* | ADORA1, CDK1, EGFR, ERBB2, GAPDH, IMPDH2, DUSP16, DOT1L., SLC29A1, ADA | EGFR, CDK1, ADORA1 | - | -1.85 |
| Inflammation | *Manis pentadactyla* | PPARG, CDC25C, ABCB1, CYP51A1, TOP2B, PPARD, ABCC4, HDAC1, HDAC2, HDAC3 | PPARG, ABCC4, HDAC2 | ABCC4, TOP2B, HDAC1 | -3.34 |
|  | *Citrus unshiu* | PPARG, CA9, CDC25C, GSK3B, CA6, GAPDH, HSP90AB1, MMP2, EGFR, CALM1 | PPARG, EGFR, HSP90AB1 | EGFR, PPARG, CALM1 | -3.38 |
|  | *Gleditsia sinensis* | STAT3, PLAU, CA9, CDC25C, RELA, APP, SRC, CDK2, CA6, PTPN2 | STAT3, EGFR, APP | STAT3, EGFR, PTPN11 | -2.59 |

Table S12. Results of the herb-level analysis for each phenotype from the literature review.

| **Phenotype** | **Herb combination** | **Matched herb targets** | **Literature evidence** |
| --- | --- | --- | --- |
| Asthma | *Morus alba* (PMID: 23806866), *Ephedra sinica* (PMID: 36301619), *Perilla frutescens* (PMID: 34045925), *Pinellia ternata* (PMID: 37268256) | ESR1 | PMID: 19433448 |
|  |  | CDK2 | PMID: 31637021 |
|  |  | PARP1 | PMID: 26205779 |
|  |  | APP | PMID: 29956778 |
| Diabetes | *Trichosanthes kirilowii* (PMID: 28100206), *Nelumbo nucifera* (PMID: 31324289), *Dioscorea polystachya* (PMID: 34521490)*, Rehmannia glutinosa* (PMID: 14698506) | RELA | PMID: 37311878 |
|  |  | PTGS2 | PMID: 37705740 |
|  |  | PPARG | PMID: 32728045 |
|  |  | PTPN11 | PMID: 31284101 |
|  |  | TOP1 | PMID: 22247452 |
|  |  | PRKCB | PMID: 18278479 |
|  |  | NR1I2 | PMID: 29309761 |
|  |  | ESR1 | PMID: 18854778 |
| Arthritis | *Gentiana macrophylla* (PMID: 15374610), *Angelica pubescens* (PMID: 32256373), *Achyranthes bidentata* (PMID: 35988424) | PTPN2 | PMID: 30620725 |
|  |  | RELA | PMID: 30894909 |
|  |  | STAT3 | PMID: 28887478 |
|  |  | SMAD3 | PMID: 36934797 |
|  |  | IGF1R | PMID: 28583713 |
|  |  | PTK2 | PMID: 36934797 |
|  |  | PTGS2 | PMID: 14671726 |
|  |  | PPARG | PMID: 19646655 |
| Stroke | *Acorus gramineus* (PMID: 36628348), *Pinellia ternata* (PMID: 25847568), *Arisaema amurense* (PMID: 34887934) | BCL2L1 | PMID: 34299322 |
|  |  | ESR2 | PMID: 22305517 |
|  |  | APP | PMID: 26519139 |
|  |  | CDK1 | PMID: 29581894 |
|  |  | MMP9 | PMID: 26973468 |
|  |  | ADORA1 | PMID: 34356346 |
|  |  | EGFR | PMID: 35862191 |
| Inflammation | *Manis pentadactyla* (PMID: 39397000),  *Citrus unshiu* (PMID: 34451753),  *Gleditsia sinensis* (PMID: 36921533) | PPARG | PMID: 38648706 |
|  |  | ABCC4 | PMID: 28659663 |
|  |  | HDAC1 | PMID: 35148729 |
|  |  | HDAC2 | PMID: 30407865 |
|  |  | EGFR | PMID: 36827922 |
|  |  | CALM1 | PMID: 38635101 |
|  |  | HSP90AB1 | PMID: 36979785 |
|  |  | STAT3 | PMID: 23483479 |
|  |  | APP | PMID: 30756214 |
|  |  | TOP2B | PMID: 38296114 |

Table S13. The overlap ratio between compounds identified using ARM and those determined using network analysis.

| **Phenotype** | **Compounds from compound–phenotype ARM** | **Compounds from compound–phenotype network analysis** | **Number of overlapping compounds** | **Szymkiewicz–Simpson coefficient^a^** |
| --- | --- | --- | --- | --- |
| Asthma | 1833 | 313 | 105 | 0.3465 |
| Diabetes | 1916 | 388 | 299 | 0.7706 |
| Arthritis | 1797 | 78 | 30 | 0.3846 |
| Stroke | 2656 | 89 | 60 | 0.6742 |
| Inflammation | 1977 | 99 | 53 | 0.5354 |

^a^ This analysis was adopted as it is suitable for comparing two datasets of different sizes [10, 11].

Table S14. Compound-level analysis results for each phenotype from the literature review.

| **Phenotype** | **Herbal material** | **Related compound** | **Literature evidence** |
| --- | --- | --- | --- |
| Asthma | *Morus alba* | D-tryptophan | PMID: 35956928 |
|  |  | Limonene | PMID: 23166622 |
|  | *Ephedra sinica* | D-tryptophan | PMID: 29327126 |
|  |  | Limonene | PMID: 26968679 |
|  | *Perilla frutescens* | D-tryptophan | PMID: 37324746 |
|  |  | Carvacrol | PMID: 35684514 |
|  |  | Limonene | PMID: 35684514 |
|  | *Pinellia ternata* | D-tryptophan | PMID: 16784905 |
| Diabetes | *Trichosanthes kirilowii* | Ferulic acid | PMID: 21958542 |
|  | *Nelumbo nucifera* | (-)-epicatechin-3-O-gallate | PMID: 35781857 |
|  |  | Docosahexaenoic acid | doi.org/10.1016/j.lwt.2024.116848 |
|  |  | Ferulic acid | PMID: 34685815 |
|  | *Dioscorea polystachya* | (-)-epicatechin-3-O-gallate | PMID: 36985850 |
|  | *Rehmannia glutinosa* | Docosahexaenoic acid | PMID: 34810292 |
|  |  | Ferulic acid | PMID: 37522376 |
| Arthritis | *Gentiana macrophylla* | Glutamic acid | PMID: 38858643 |
|  |  | EtOAc | PMID: 22475010 |
|  | *Angelica pubescens* | EtOAc | PMID: 20371279 |
|  |  | Scopoletin | PMID: 20183310 |
|  | *Achyranthes bidentata* | Glutamic acid | PMID: 15503770 |
|  |  | Scopoletin | PMID: 38930905 |
| Stroke | *Acorus gramineus* | Choline | PMID: 20673844 |
|  |  | β-sitosterol | PMID: 11105577 |
|  |  | Adenosine | PMID: 15187444 |
|  | *Pinellia ternata* | Choline | PMID: 38058630 |
|  |  | β-sitosterol | PMID: 38058630 |
|  |  | Adenosine | PMID: 38058630 |
|  | *Arisaema amurense* | Choline | doi.org/10.1016/j.jep.2021.114798 |
|  |  | β-sitosterol | doi.org/10.1016/j.jep.2021.114798 |
| Inflammation | *Manis pentadactyla* | - | - |
|  | *Citrus unshiu* | Hesperidin | PMID: 39769483 |
|  |  | Hesperetin | PMID: 36678190 |
|  |  | Linalool | PMID: 25443842 |
|  | *Gleditsia sinensis* | Hesperidin | PMID: 36921533 |
|  |  | Hesperetin | PMID: 36921533 |
|  |  | Linalool | doi.org/10.1016/j.arabjc.2022.103859 |

Table S15. Compound-level analysis results for each phenotype from the literature review.

| **Phenotype** | **Compound^a^** | **Identified compound-related herb targets** | **Literature evidence** |
| --- | --- | --- | --- |
| Asthma | D-tryptophan | MMP3 | PMID: 10377203 |
|  | (PMID: 27670239) | SLC7A5 | PMID: 37895302 |
|  |  | CA9 | PMID: 34446467 |
|  | Carvacrol | ESR1 | PMID: 19433448 |
|  | (PMID: 33773189) | JUN | PMID: 16865089 |
|  |  | BCL2 | PMID: 10770817 |
|  | Limonene | TOP2A | PMID: 34497339 |
|  | (PMID: 32789792) | EPAS1 | PMID: 36550577 |
|  |  | ESR1 | - |
|  |  | PTPN1 | PMID: 22156494 |
| Diabetes | (-)-epicatechin-3-O-gallate | EP300 | PMID: 24428157 |
|  | (PMID: 32485837) | APP | PMID: 19931251 |
|  |  | CFTR | PMID: 33659254 |
|  |  | RELA | PMID: 37311878 |
|  | Docosahexaenoic acid | EP300 | - |
|  | (PMID: 25356177) | FASN | PMID: 19181734 |
|  |  | CFTR | - |
|  |  | RELA | - |
|  | Ferulic acid | EGFR | PMID: 36359813 |
|  | (PMID: 26201855) | ESR2 | PMID: 33430527 |
|  |  | ESR1 | PMID: 18854778 |
|  |  | RELA | - |
| Arthritis | Glutamic acid | GSK3B | PMID: 35747513 |
|  | (PMID: 26521747) | RELA | PMID: 30894909 |
|  |  | AKT1 | PMID: 20187155 |
|  | EtOAc | APP | PMID: 38474288 |
|  | (PMID: 14648391) | PTPN1 | PMID: 24590766 |
|  |  | RELA | - |
|  | Scopoletin | APP | - |
|  | (PMID: 19845767) | EGFR | PMID: 33441426 |
|  |  | GAPDH | PMID: 24493325 |
| Stroke | Choline | MMP2 | PMID: 24304146 |
|  | (PMID: 26567726) | EGFR | PMID: 35862191 |
|  |  | ESR2 | PMID: 22305517 |
|  |  | APP | PMID: 26519139 |
|  | Adenosine | EGFR | - |
|  | (PMID: 20190963) | ADORA1 | PMID: 34356346 |
|  |  | ERBB2 | PMID: 23403761 |
|  | β-sitosterol | MMP2 | - |
|  | (PMID: 37721296) | APP | - |
|  |  | ESR2 | - |
|  |  | EGFR | - |
| Inflammation | Hesperidin | PPARG | PMID: 38648706 |
|  | (PMID: 36736168) | EGFR | PMID: 36827922 |
|  |  | APP | PMID: 30756214 |
|  | Hesperetin | PPARG | - |
|  | (PMID: 33068864) | AKT1 | PMID: 19622728 |
|  |  | DPP4 | PMID: 32523042 |
|  |  | GSK3B | PMID: 31978503 |
|  |  | RELA | PMID: 33068864 |
|  | Linalool | PPARG | - |
|  | (PMID: 31254955) | SRC | PMID: 31918290 |
|  |  | EGFR | - |

^a^This literature search used PubMed to identify compounds known to be biologically relevant to the target phenotype and selected compounds with direct experimental evidence for their efficacy. This process prioritized compounds identified in the DrugBank as clinically tested for phenotype treatment, whereas compounds used as placebos were excluded.

Table S16. Structural network analysis results and biological pathway searches for each phenotype.

| **Phenotype** | **Selected compounds in the literature review** | **Hub of the network** | **Bottleneck of the network** | **Related biological pathways** |
| --- | --- | --- | --- | --- |
| Diabetes | (-)-epicatechin-3-O-gallate | EP300, APP, CFTR | RELA | Type II diabetes mellitus, insulin signaling pathway, maturity-onset diabetes of the young, apoptosis, PI3K–AKT signaling pathway, insulin secretion, other pathways, and genetic evidence-based genes |
|  | Docosahexaenoic acid | EP300, FASN, CFTR | RELA | Insulin signaling pathway, maturity-onset diabetes of the young, apoptosis, PI3K–AKT signaling pathway, insulin secretion, other pathways, and genetic evidence-based genes |
|  | Ferulic acid | EGFR, ESR2, ESR1 | RELA | Insulin signaling pathway, apoptosis, PI3K–AKT signaling pathway, insulin secretion, other pathways, and genetic evidence-based genes |
| Arthritis | Glutamic acid | GSK3B, RELA, AKT1 | - | Regulation of the actin cytoskeleton, MAPK signaling pathway, osteoclast differentiation, leukocyte transendothelial migration, T cell receptor signaling pathway, other pathways, and genetic evidence-based genes |
|  | EtOAc | APP, RELA, PTPN1 | - | Rheumatoid arthritis, regulation of the actin cytoskeleton, MAPK signaling pathway, osteoclast differentiation, leukocyte transendothelial migration, T cell receptor signaling pathway, other pathways, and genetic evidence-based genes |
|  | Scopoletin | EGFR, APP, GAPDH | GAPDH | Insulin signaling pathway, apoptosis, PI3K–AKT signaling pathway, insulin secretion, other pathways, and genetic evidence-based genes |
| Stroke | Choline | APP, EGFR, ESR2 | MMP2 | Lipid and atherosclerosis, PI3K–AKT signaling pathway, apoptosis, other pathways, and genetic evidence-based genes |
|  | β-sitosterol | APP, EGFR, ESR2 | MMP2 | Rheumatoid arthritis, regulation of the actin cytoskeleton, MAPK signaling pathway, osteoclast differentiation, leukocyte transendothelial migration, T cell receptor signaling pathway, other pathways, and genetic evidence-based genes  Lipid and atherosclerosis, PI3K–AKT signaling pathway, other pathways, and genetic evidence-based genes |
|  | Adenosine | EGFR, ERBB2, ADORA1 | - |  |
| Inflammation | Hesperidin | PPARG, EGFR, APP | PPARG, EGFR, APP | Chemokine signaling pathway, cytokine–cytokine receptor interaction, MAPK signaling pathway, NF-kappa B signaling pathway, other pathways, and genetic evidence-based genes |
|  | Hesperetin | PPARG, AKT1, DPP4 | PPARG, GSK3B, RELA |  |
|  | Linalool | PPARG, SRC, EGFR | PPARG, SRC, EGFR | Lipid and atherosclerosis, PI3K–AKT signaling pathway, other pathways, and genetic evidence-based genes |

Table S17. The literature review results for pathway information within hubs and bottlenecks of compounds for each phenotype.

| **Phenotype** | **Selected compounds using the literature review** | **Hubs or bottlenecks in the network** | **Biological pathways associated with the target** |
| --- | --- | --- | --- |
| Asthma | D-tryptophan | CA9 (PMID: 5794225) | Cell adhesion molecules (PMID: 32824856) |
|  | (PMID: 27670239) | MMP3 (PMID: 32867828) | NF-kappa B signaling pathway (PMID: 32867828) |
|  |  |  | Cytokine–cytokine receptor interaction (PMID: 26851968) |
|  |  | SLC7A5 (PMID: 39512508) | NF-kappa B signaling pathway (PMID: 34869729) |
|  | Carvacrol | BCL2 (PMID: 26214321) | PI3K–AKT signaling pathway (PMID: 39381113) |
|  | (PMID: 33773189) |  | NF-kappa B signaling pathway (PMID: 33516930) |
|  |  |  | Cell adhesion molecules (PMID: 20142842) |
|  |  | ESR1 (PMID: 33571611) | PI3K–AKT signaling pathway (PMID: 19420388) |
|  |  |  | NF-kappa B signaling pathway (PMID: 19433448) |
|  |  |  | T cell receptor signaling pathway (PMID: 29666308) |
|  |  | JUN (PMID: 26214321) | Other pathways, and genetic evidence-based genes (PMID: 19099816) |
|  | Limonene | ESR1 (PMID: 36358576) | PI3K-AKT signaling pathway (PMID: 19420388) |
|  | (PMID: 32789792) |  | NF-kappa B signaling pathway (PMID: 19433448) |
|  |  |  | T cell receptor signaling pathway (PMID: 29666308) |
|  |  | EPAS1 (PMID: 34033902) | Cell adhesion molecules (PMID: 36325096) |
|  |  | TOP2A (PMID: 39259370) | NF-kappa B signaling pathway (PMID: 36138481) |
|  |  |  | Cytokine–cytokine receptor interaction (PMID: 38661103) |
|  |  | PTPN1 (PMID: 32595497) | T cell receptor signaling pathway (PMID: 37982351) |
|  |  |  | Cytokine–cytokine receptor interaction (PMID: 38707187) |
|  |  |  | Other pathways, and genetic evidence-based genes (PMID: 23590304) |
| Diabetes | (-)-epicatechin-3-O-gallate | APP (PMID: 33137558) | PI3K–AKT signaling pathway (PMID: 34830036) |
|  | (PMID: 32485837) |  | Insulin signaling pathway (PMID: 27392857) |
|  |  |  | Apoptosis (PMID: 32089787) |
|  |  | CFTR (PMID: 25747701) | Type II diabetes mellitus (PMID: 34825893) |
|  |  |  | PI3K–AKT signaling pathway (PMID: 28213469) |
|  |  |  | Insulin secretion (PMID: 28977592) |
|  |  | EP300 (PMID: 28194107) | Maturity onset diabetes of the young (PMID: 11435618) |
|  |  |  | Other pathways, and genetic evidence-based genes (PMID: 27121852) |
|  |  | RELA (PMID: 33038311) | Insulin secretion (PMID: 37311878) |
|  |  |  | Apoptosis (PMID: 10085110) |
|  | Docosahexaenoic acid | CFTR (PMID: 19209467) | Type II diabetes mellitus (PMID: 34825893) |
|  | (PMID: 25356177) |  | PI3K–AKT signaling pathway (PMID: 28213469) |
|  |  |  | Insulin secretion (PMID: 28977592) |
|  |  | EP300 (PMID: 35504444) | Maturity onset diabetes of the young (PMID: 11435618) |
|  |  |  | Other pathways, and genetic evidence-based genes (PMID: 27121852) |
|  |  | FASN (PMID: 37224334) | Insulin signaling pathway (PMID: 30274245) |
|  |  |  | Apoptosis (PMID: 34620841) |
|  |  | RELA (PMID: 30622239) | Insulin secretion (PMID: 37311878) |
|  |  |  | Apoptosis (PMID: 10085110) |
|  | Ferulic acid | EGFR (PMID: 29164418) | PI3K–AKT signaling pathway (PMID: 36359813) |
|  | (PMID: 26201855) |  | Other pathways, and genetic evidence-based genes (PMID: 39205643) |
|  |  | ESR1 (PMID: 16893382) | Insulin signaling pathway (PMID: 33749878) |
|  |  | ESR2 (PMID: 37517566) | PI3K–AKT signaling pathway (PMID: 19211725) |
|  |  |  | Apoptosis (PMID: 37967683) |
|  |  | RELA (PMID: 39519712) | Insulin secretion (PMID: 37311878) |
|  |  |  | Apoptosis (PMID: 10085110) |
| Arthritis | Glutamic acid | AKT1 (PMID: 22505016) | Regulation of the actin cytoskeleton (PMID: 19303207) |
|  | (PMID: 26521747) |  | MAPK signaling pathway (PMID: 38253276) |
|  |  |  | T cell receptor signaling pathway (PMID: 10891487) |
|  |  | GSK3B (PMID: 25454285) | Osteoclast differentiation (PMID: 28731198) |
|  |  |  | Leukocyte transendothelial migration (PMID: 22863953) |
|  |  | RELA (PMID: 20623531) | Osteoclast differentiation (PMID: 18464930) |
|  |  |  | Other pathways, and genetic evidence-based genes (PMID: 38794813) |
|  | EtOAc | APP (PMID: 30736391) | Osteoclast differentiation (PMID: 23649480) |
|  | (PMID: 11801382) |  | Leukocyte transendothelial migration (PMID: 27444968) |
|  |  | RELA (PMID: 31906558) | Osteoclast differentiation (PMID: 18464930) |
|  |  |  | Other pathways, and genetic evidence-based genes (PMID: 38794813) |
|  |  | PTPN1 (PMID: 25598795) | Regulation of the actin cytoskeleton (PMID: 12902327) |
|  |  |  | Osteoclast differentiation (PMID: 34812548) |
|  |  |  | Other pathways, and genetic evidence-based genes (PMID: 39298571) |
|  | Scopoletin | APP (PMID: 32671342) | Osteoclast differentiation (PMID: 23649480) |
|  | (PMID: 19845767) |  | Leukocyte transendothelial migration (PMID: 27444968) |
|  |  | EGFR (PMID: 35378275) | Rheumatoid arthritis (PMID: 38789573) |
|  |  |  | Regulation of the actin cytoskeleton (PMID: 28634045) |
|  |  |  | MAPK signaling pathway (PMID: 36178273) |
|  |  | GAPDH (PMID: -) | Osteoclast differentiation (PMID: 37973217) |
|  |  |  | T cell receptor signaling pathway (PMID: 31447347) |
| Stroke | Choline | APP (PMID: 37548694) | Lipid and atherosclerosis (PMID: 19945109) |
|  | (PMID: 26567726) |  | PI3K–AKT signaling pathway (PMID: 34830036) |
|  |  |  | Apoptosis (PMID: 32089787) |
|  |  | EGFR (PMID: 30521373) | PI3K–AKT signaling pathway (PMID: 24493201) |
|  |  |  | Other pathways, and genetic evidence-based genes (PMID: 37935323) |
|  |  | ESR2 (PMID: 22156442) | PI3K–AKT signaling pathway (PMID: 19211725) |
|  |  |  | Apoptosis (PMID: 37967683) |
|  |  | MMP2 (PMID: 39273042) | Apoptosis (PMID: 39538054) |
|  | β-sitosterol | APP (PMID: 31908573) | Lipid and atherosclerosis (PMID: 19945109) |
|  | (PMID: 37721296) |  | PI3K–AKT signaling pathway (PMID: 34830036) |
|  |  |  | Apoptosis (PMID: 32089787) |
|  |  | EGFR (PMID: 35807354) | PI3K–AKT signaling pathway (PMID: 24493201) |
|  |  |  | Other pathways and genetic evidence-based genes (PMID: 37935323) |
|  |  | ESR2 (PMID: 15113961) | PI3K–AKT signaling pathway (PMID: 19211725) |
|  |  |  | Apoptosis (PMID: 37967683) |
|  |  | MMP2 (PMID: 31473743) | Apoptosis (PMID: 39538054) |
|  | Adenosine | ADORA1 (PMID: 37081178) | PI3K–AKT signaling pathway (PMID: 33293832) |
|  | (PMID: 20190963) |  | Other pathways and genetic evidence-based genes (PMID: 34356346) |
|  |  | EGFR (PMID: 19574994) | PI3K–AKT signaling pathway (PMID: 24493201) |
|  |  |  | Other pathways and genetic evidence-based genes (PMID: 37935323) |
|  |  | ERBB2 (PMID: 18799465) | Lipid and atherosclerosis (PMID: 32160892) |
| Inflammation | Hesperidin | PPARG (PMID: 30623541) | Chemokine signaling pathway (PMID: 39094710) |
|  | (PMID: 36736168) |  | Cytokine–cytokine receptor interaction (PMID: 34638771) |
|  |  |  | Other pathways and genetic evidence-based genes (PMID: 37358659) |
|  |  | EGFR (PMID: 39781340 | NF-kappa B signaling pathway (PMID: 26718225) |
|  |  | APP (PMID: 25510196) | NF-kappa B signaling pathway (PMID: 35406030) |
|  |  |  | MAPK signaling pathway (PMID: 32475008) |
|  | Hesperetin | PPARG (PMID: 33880358) | Chemokine signaling pathway (PMID: 39094710) |
|  | (PMID: 33068864) |  | Cytokine–cytokine receptor interaction (PMID: 34638771) |
|  |  |  | Other pathways and genetic evidence-based genes (PMID: 37358659) |
|  |  | AKT1 (PMID: 34262419) | MAPK signaling pathway (PMID: 38253276) |
|  |  |  | Other pathways and genetic evidence-based genes (PMID: 38613881) |
|  |  | DPP4 (PMID: 35700199) | NF-kappa B signaling pathway (PMID: 38424257) |
|  |  | GSK3B (PMID: 25454285) | Chemokine signaling pathway (PMID: 24033914) |
|  | Linalool | PPARG (PMID: 33880358) | Chemokine signaling pathway (PMID: 39094710) |
|  | (PMID: 31254955) |  | Cytokine–cytokine receptor interaction (PMID: 34638771) |
|  |  |  | Other pathways and genetic evidence-based genes (PMID: 37358659) |
|  |  | SRC (PMID: 27185319) | MAPK signaling pathway (PMID: 16530387) |
|  |  | EGFR (PMID: 39781340) | NF-kappa B signaling pathway (PMID: 26718225) |

**B. The compound-level analysis and literature review results for five phenotypes**

**1. Asthma**

In total, 16 compounds were found among both the top 150 compounds from the ARM analysis and the 313 herbal compounds from the network analysis; all 16 were proximal to the phenotype. Furthermore, of these 16 compounds, D-tryptophan, carvacrol, and limonene were identified as effective compounds in the literature review, known to alleviate allergic airway diseases or exhibit anti-inflammatory effects [12-14]. We conducted a structural network analysis and a literature review to analyze the molecular mechanisms involving these three identified compounds; the results showed that the identified compound targets were associated with multiple asthma-related pathways. Carvacrol has a high binding affinity for the estrogen receptor alpha, which is encoded by ESR1 [15]. ESR1 is associated with several other pathways associated with asthma, including the PI3K–AKT, NF-kappa B, and T cell receptor signaling pathways [16-21]. These associations act both singularly and through interactions with other genes. ESR1 genetic variants are associated with asthma in female patients [22]. In addition, although the direct relationship between limonene and TOP2A has not been studied, increased TOP2A expression is known to promote prostate signaling, while limonene breakdown in prostate cancer patients has a protective effect [23]. DNA topoisomerase II alpha, which TOP2A encodes, is related to the NF-kappa B signaling pathway and was identified as one of the genes upregulated in airway epithelial cell transcriptome analysis for asthma [24-27].

**2. Diabetes**

In total, 5 of the top 150 compounds from the ARM analysis and 388 herbal compounds from the network analysis overlapped; all were proximal to the phenotype. Among these five, three effective compounds were identified through the literature review: (-)-epicatechin-3-O-gallate, docosahexaenoic acid, and ferulic acid. Structural network analysis was performed to determine the target interactions of these compounds and any associated biological pathways relevant to diabetes (Fig. S1). Rats administered a green tea extract containing (-)-epicatechin-3-O-gallate exhibited significantly reduced high blood glucose levels [28]. Docosahexaenoic acid shows antidiabetic effects in streptozotocin-induced diabetic mice [29]. Ferulic acid helps maintain normal glucose homeostasis by inhibiting gluconeogenesis and negative regulators of insulin signaling in type 2 diabetic rats [30]. The structural network analysis showed that the compound targets we identified as hubs and bottlenecks were associated with multiple diabetes-related pathways. For example, docosahexaenoic acid induces the expression of fatty acid synthase (FASN); meanwhile, FASN inhibition is known to improve glucose metabolism and insulin sensitivity [31, 32]. Ferulic acid has been shown to inhibit the epidermal growth factor receptor (EGFR) activation in breast cancer cells *in vitro* [33]. EGFR inhibition can help alleviate diabetic complications, such as diabetic nephropathy and diabetic cardiomyopathy, through the PI3K–AKT pathway suppression [34].


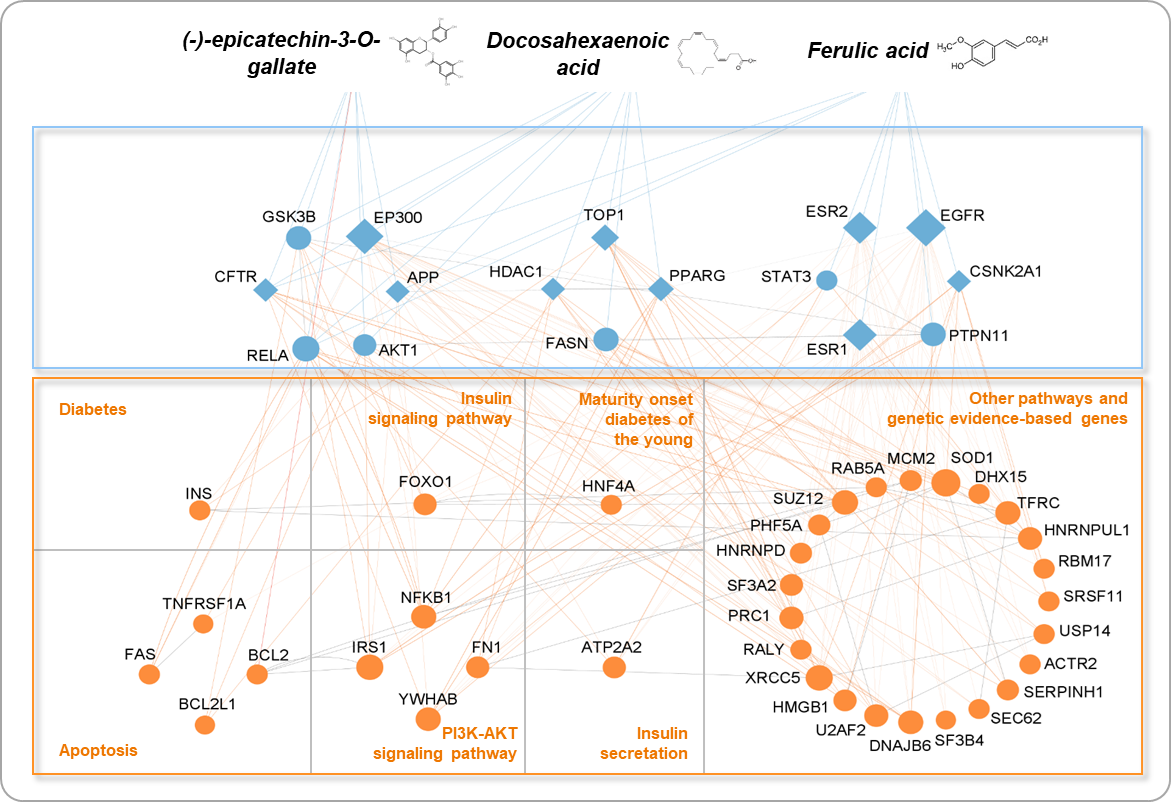


Figure S1. Structural network analysis results based on the therapeutic potential of the three identified compounds, (-)-epicatechin-3-O-gallate, docosahexaenoic acid, and ferulic acid, in diabetes. Structural network analysis categorized nodes into compound targets (blue) and phenotype-related genes (orange). Nodes with compound targets and phenotype-related genes are represented by circular nodes instead of the rhombus shape used for other compound targets. Edges are colored to indicate the type of interaction. Orange edges represent interactions between compound targets and phenotype-related genes, while red edges indicate interactions between compounds and phenotype-related genes. Phenotype-related genes are classified into seven categories based on the biological pathway to which they belong.

**3. Arthritis**

In total, 9 of the top 150 compounds from the ARM analysis and 78 herbal compounds from the network analysis overlapped; all were proximal to the phenotype. Among these nine, three effective compounds were identified through the literature review: glutamic acid, ethyl acetate (EtOAc), and scopoletin. Further, structural network analysis and the literature review revealed an association between the identified compound targets and several arthritis-related pathways (Fig. S2). Glutamate, the amino acid derivative of glutamic acid, plays a crucial role in inflammatory responses and regulates osteoarthritis via membrane receptors, such as NMDA [35]. The active ingredient extracted from *Kalopanax pictus* using EtOAc has shown significant anti-arthritis effects compared to other fractions [36]. Additionally, scopoletin can alleviate clinical symptoms in supplement-induced arthritic rats, notably by reducing the production of endogenous angiogenesis inducers [37]. The compound targets we identified as hubs and bottlenecks were found to interact with multiple pathways involved in the phenotype, including cytoskeleton regulation, MAPK signaling pathway, and osteoclast differentiation. For example, the beneficial effects of scopoletin have been shown to result from MAPK signaling pathway modulation, likely due to its interaction with epidermal growth factor receptor (EGFR), which, upon ligand binding, activates downstream pathways including the MAPK signaling pathway [38, 39].


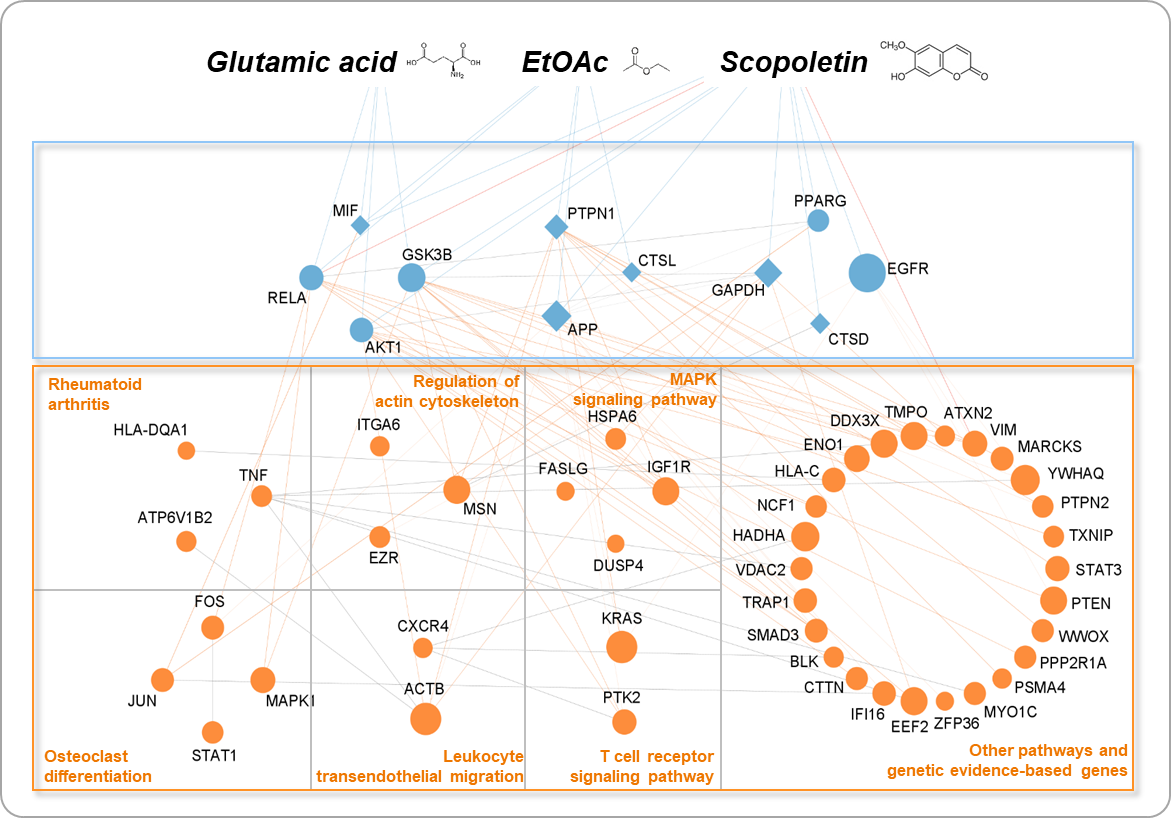


Figure S2. Structural network analysis results based on the therapeutic potential of the three identified compounds, glutamic acid, EtOAc, and scopoletin, in arthritis. Structural network analysis categorized nodes into compound targets (blue) and phenotype-related genes (orange). Nodes with compound targets and phenotype-related genes are represented by circular nodes instead of the rhombus shape used for other compound targets. Edges are colored to indicate the type of interaction. Orange edges represent interactions between compound targets and phenotype-related genes, while red edges indicate interactions between compounds and phenotype-related genes. Phenotype-related genes are classified into seven categories based on the biological pathway to which they belong.

**4. Stroke**

In total, 22 of the top 150 compounds from the ARM analysis and 89 herbal compounds from the network analysis overlapped; all were proximal to the phenotype. Of the 22 compounds, the literature review identified three as effective: choline, adenosine, and β-sitosterol. The structural network analysis and literature review showed that the targets of the identified compounds were involved in multiple stroke-related pathways (Fig. S3). Oral choline administration significantly improves neurological deficits, reduced infarct volume, and prevented neuronal cell loss in the ischemic cerebral cortex of permanent middle cerebral artery occlusion rats [40]. Meanwhile, modulating adenosine receptors influenced infarct volume in animal models, with evidence suggesting that specific adenosine receptor activation likely triggers an anti-inflammatory response from immune cells during stroke [41]. Additionally, β-sitosterol may aid ischemic stroke treatment by inhibiting pathways related to neuronal intracellular cholesterol overload and endoplasmic reticulum stress [42]. The identified compound targets, which act as hubs and bottlenecks, interact with pathways linked to phenotypes such as lipid and atherosclerosis, PI3K–AKT, and apoptosis signaling pathways. For example, adenosine is known to activate ERBB2, which is associated with lipid and atherosclerosis, and the PI3K–AKT signaling pathway via ADORA1 [43-45].


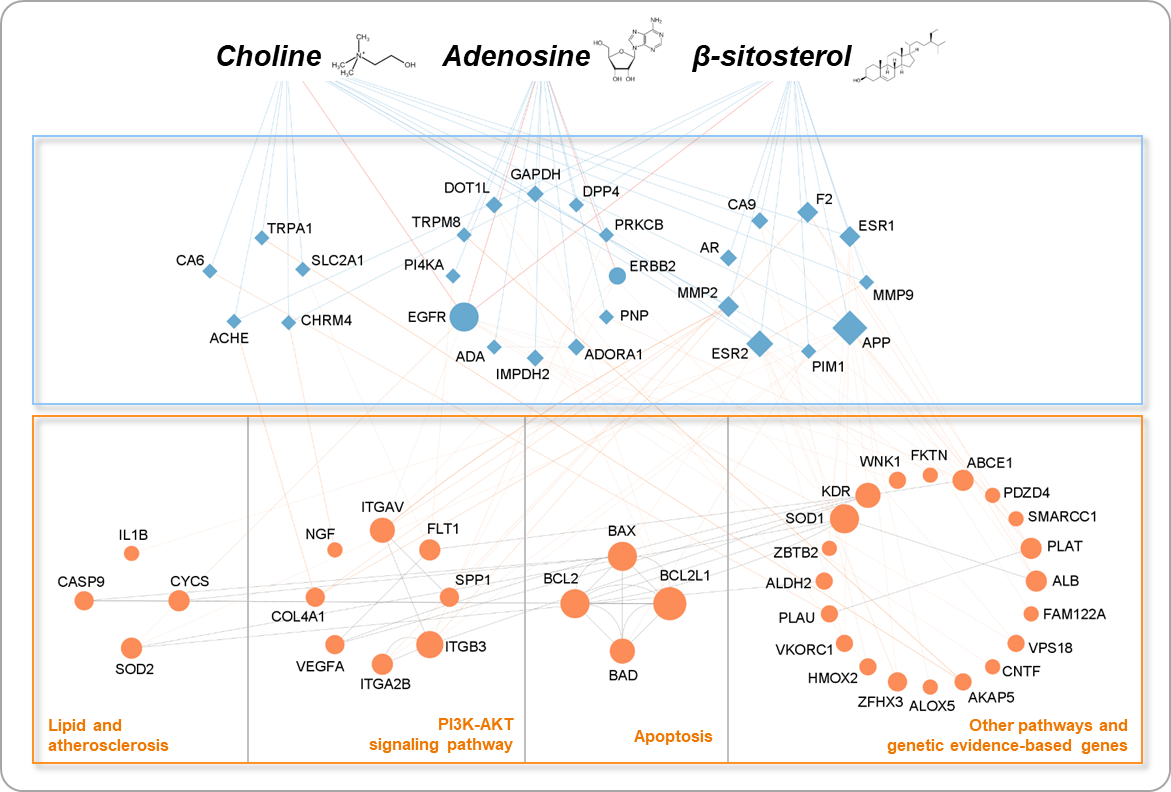


Figure S3. Structural network analysis results based on the therapeutic potential of the three identified compounds, choline, adenosine, and β-sitosterol, in stroke. Structural network analysis categorized nodes into compound targets (blue) and phenotype-related genes (orange). Nodes with compound targets and phenotype-related genes are represented by circular nodes instead of the rhombus shape used for other compound targets. Edges are colored to indicate the type of interaction. Orange edges represent interactions between compound targets and phenotype-related genes, while red edges indicate interactions between compounds and phenotype-related genes. Phenotype-related genes are classified into four categories based on the biological pathway to which they belong.

**5. Inflammation**

In total, 11 of the top 150 compounds from the ARM analysis and 99 herbal compounds from the network analysis overlapped; all were proximal to the phenotype. Among these 11, three effective compounds were identified through the literature review: hesperidin, hesperetin, and linalool. The structural network analysis and literature review indicated that the targets of the identified compounds are associated with various inflammation-related pathways (Fig. S4). Hesperidin has been shown to inhibit the IL6/STAT3 signaling pathway, suggesting the potential use of hesperidin in treating pulmonary fibrosis [46]. A previous study indicates that hesperetin acts as an SIRT1 activator, making it a promising therapeutic candidate for inflammatory diseases [47]. Linalool has effectively reduced airway inflammation and mucus hypersecretion induced by ovalbumin [48]. The identified compound targets, acting as hubs and bottlenecks, are involved in multiple pathways relevant to the phenotype, including chemokine signaling, cytokine–cytokine receptor interactions, and NF-kappa B signaling. For instance, hesperetin combination therapy protects against LPS-induced neuroinflammation by reducing proinflammatory cytokine expression and modulating the NF-kappa B signaling pathway. This effect may result from interactions, such as RELA inhibition, driven by SIRT1 expression [47, 49].


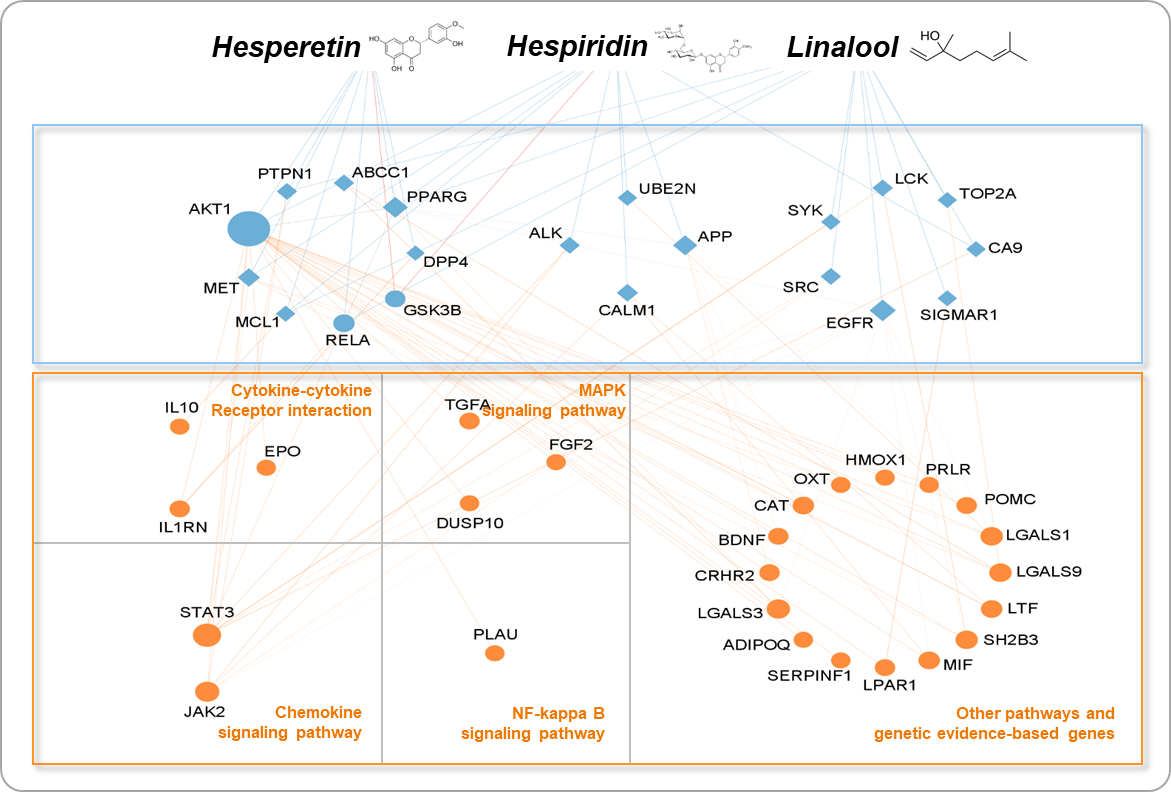


Figure S4. Structural network analysis results based on the therapeutic potential of the three identified compounds, hesperetin, hesperidin, and linalool, in stroke. Structural network analysis categorized nodes into compound targets (blue) and phenotype-related genes (orange). Nodes with compound targets and phenotype-related genes are represented by circular nodes instead of the rhombus shape used for other compound targets. Edges are colored to indicate the type of interaction. Orange edges represent interactions between compound targets and phenotype-related genes, while red edges indicate interactions between compounds and phenotype-related genes. Phenotype-related genes are classified into five categories based on the biological pathway to which they belong.


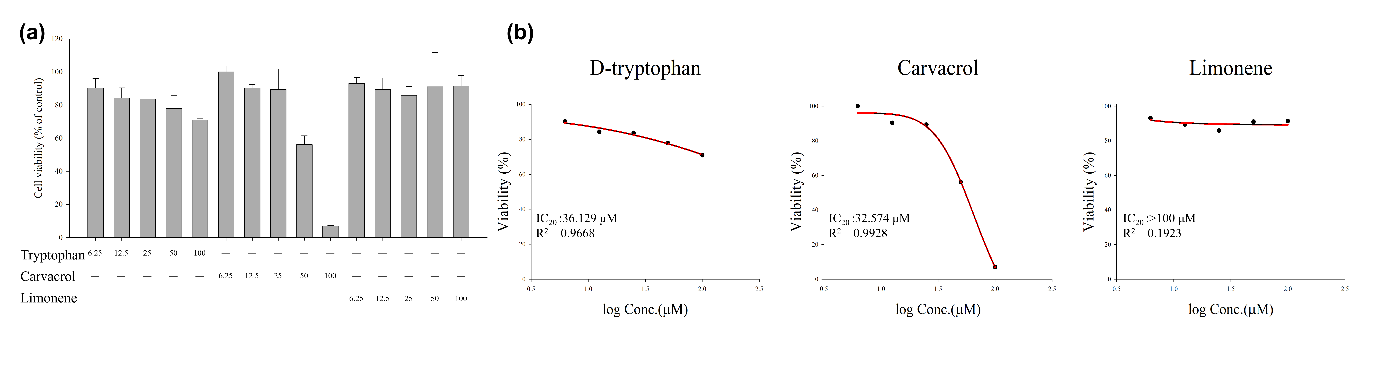


Figure S5. (a) Measured cell viability and (b) IC_20_ values were used to parameterize concentration–effect curves to determine the appropriate treatment concentration.


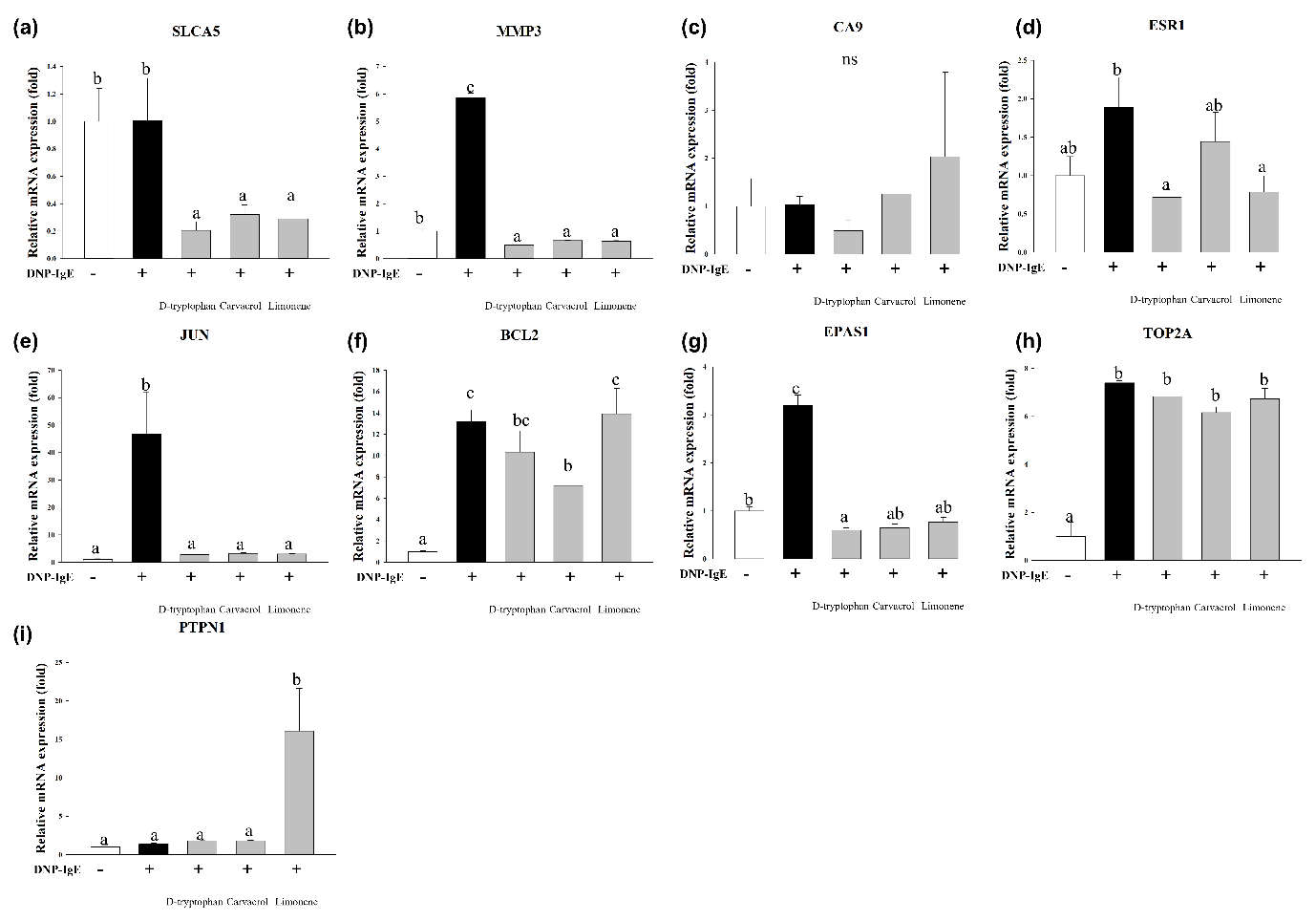


Figure S6. Validation of the effects on hub and bottleneck target genes associated with the structural network analysis for asthma. The letters above the bars indicate significant differences (Duncan’s multiple range test; *p* < 0.05). The order of values from small to large is alphabetical.

**References**

1. Federhen S. The NCBI taxonomy database, Nucleic Acids Res 2012;40:D136–D143.

2. Kim S, Thiessen PA, Bolton EE et al. PubChem substance and compound databases, Nucleic Acids Res 2016;44:D1202–D1213.

3. Gaulton A, Bellis LJ, Bento AP et al. ChEMBL: a large-scale bioactivity database for drug discovery, Nucleic Acids Res 2012;40:D1100–D1107.

4. Maglott D, Ostell J, Pruitt KD et al. Entrez gene: gene-centered information at NCBI, Nucleic Acids Res 2011;39:D52–D57.

5. Aronson AR, Lang FM. An overview of MetaMap: historical perspective and recent advances, J Am Med Inform Assoc 2010;17:229–236.

6. Bodenreider O. The unified medical language system (UMLS): integrating biomedical terminology, Nucleic Acids Res 2004;32:D267–D270.

7. Kohler S, Gargano M, Matentzoglu N et al. The human phenotype ontology in 2021, Nucleic Acids Res 2021;49:D1207–D1217.

8. Agrawal R, Imieliński T, Swami A. Mining association rules between sets of items in large databases. In: Proceedings of the 1993 ACM SIGMOD international conference on Management of data (MOD). 1993, p. 207–216.

9. Hofmann H, Wilhelm A. Visual comparison of association rules, Comput Stat 2001;16:399–415.

10. Wald J, Dhamo H, Navab N et al. Learning 3d semantic scene graphs from 3d indoor reconstructions. In: Proceedings of the IEEE/CVF Conference on Computer Vision and Pattern Recognition (CVPR). 2020, p. 3961–3970.

11. Vijaymeena M, Kavitha K. A survey on similarity measures in text mining, MLAIJ 2016;3:19–28.

12. Ghorani V, Alavinezhad A, Rajabi O et al. Carvacrol improves pulmonary function tests, oxidant/antioxidant parameters and cytokine levels in asthmatic patients: a randomized, double-blind, clinical trial, Phytomedicine 2021;85:153539.

13. Kepert I, Fonseca J, Muller C et al. D-tryptophan from probiotic bacteria influences the gut microbiome and allergic airway disease, J Allergy Clin Immunol 2017;139:1525–1535.

14. Patel M, Narke D, Kurade M et al. Limonene-induced activation of A(2A) adenosine receptors reduces airway inflammation and reactivity in a mouse model of asthma, Purinergic Signal 2020;16:415–426.

15. Zhang X, Peng Y, Wu C. Chicken embryonic toxicity and potential *in vitro* estrogenic and mutagenic activity of carvacrol and thymol in low dose/concentration, Food Chem Toxicol 2021;150:112038.

16. Kazi AA, Molitoris KH, Koos RD. Estrogen rapidly activates the PI3K/AKT pathway and hypoxia-inducible factor 1 and induces vascular endothelial growth factor A expression in luminal epithelial cells of the rat uterus, Biol Reprod 2009;81:378–387.

17. Mohammad I, Starskaia I, Nagy T et al. Estrogen receptor alpha contributes to T cell-mediated autoimmune inflammation by promoting T cell activation and proliferation, Sci Signal 2018;11.

18. Zhao Y, Li X, Xu Z et al. PI3K-AKT-mTOR signaling pathway: the intersection of allergic asthma and cataract, Pharmazie 2019;74:598–600.

19. Wang X, Belguise K, O'Neill CF et al. RelB NF-kappaB represses estrogen receptor alpha expression via induction of the zinc finger protein Blimp1, Mol Cell Biol 2009;29:3832–3844.

20. Colgan JD, Hankel IL. Signaling pathways critical for allergic airway inflammation, Curr Opin Allergy Clin Immunol 2010;10:42–47.

21. Edwards MR, Bartlett NW, Clarke D et al. Targeting the NF-kappaB pathway in asthma and chronic obstructive pulmonary disease, Pharmacol Ther 2009;121:1–13.

22. Dijkstra A, Howard TD, Vonk JM et al. Estrogen receptor 1 polymorphisms are associated with airway hyperresponsiveness and lung function decline, particularly in female subjects with asthma, J Allergy Clin Immunol 2006;117:604–611.

23. Che L, Li D, Wang J et al. Identification of circadian clock-related immunological prognostic index and molecular subtypes in prostate cancer, Discov Oncol 2024;15:429.

24. Gautam Y, Johansson E, Mersha TB. Multi-omics profiling approach to asthma: an evolving paradigm, J Pers Med 2022;12.

25. Meyer-Ficca ML, Lonchar JD, Ihara M et al. Poly(ADP-ribose) polymerases PARP1 and PARP2 modulate topoisomerase II beta (TOP2B) function during chromatin condensation in mouse spermiogenesis, Biol Reprod 2011;84:900–909.

26. Hassa PO, Hottiger MO. A role of poly (ADP-ribose) polymerase in NF-kappaB transcriptional activation, Biol Chem 1999;380:953–959.

27. Fu H, Tan W, Chen Z et al. TOP2A deficit-induced abnormal decidualization leads to recurrent implantation failure via the NF-kappaB signaling pathway, Reprod Biol Endocrinol 2022;20:142.

28. Marquez Campos E, Jakobs L, Simon MC. Antidiabetic effects of flavan-3-ols and their microbial metabolites, Nutrients 2020;12.

29. Li P, Zhang L, Tian X et al. Docosahexaenoic acid has an anti-diabetic effect in streptozotocin-induced diabetic mice, Int J Clin Exp Med 2014;7:3021–3029.

30. Narasimhan A, Chinnaiyan M, Karundevi B. Ferulic acid exerts its antidiabetic effect by modulating ddsssinsulin-signalling molecules in the liver of high-fat diet and fructose-induced type-2 diabetic adult male rat, Appl Physiol Nutr Metab 2015;40:769–781.

31. Wu J, Luo J, He Q et al. Docosahexaenoic acid alters lipid metabolism processes via H3K9ac epigenetic modification in dairy goat, J Agric Food Chem 2023;71:8527–8539.

32. Zhang W, Huang J, Tang Y et al. Inhibition of fatty acid synthase (FASN) affects the proliferation and apoptosis of HepG2 hepatoma carcinoma cells via the beta-catenin/C-myc signaling pathway, Ann Hepatol 2020;19:411–416.

33. Sudhagar S, Sathya S, Anuradha R et al. Inhibition of epidermal growth factor receptor by ferulic acid and 4-vinylguaiacol in human breast cancer cells, Biotechnol Lett 2018;40:257–262.

34. Li X, Wu J, Xu F et al. Use of ferulic acid in the management of diabetes mellitus and its complications, Molecules 2022;27.

35. Wen ZH, Chang YC, Jean YH. Excitatory amino acid glutamate: role in peripheral nociceptive transduction and inflammation in experimental and clinical osteoarthritis, Osteoarthritis Cartilage 2015;23:2009–2016.

36. Choi J, Huh K, Kim SH et al. Antinociceptive and anti-rheumatoidal effects of *Kalopanax pictus* extract and its saponin components in experimental animals, J Ethnopharmacol 2002;79:199–204.

37. Pan R, Gao XH, Li Y et al. Anti-arthritic effect of scopoletin, a coumarin compound occurring in *Erycibe obtusifolia* Benth stems, is associated with decreased angiogenesis in synovium, Fundam Clin Pharmacol 2010;24:477–490.

38. Gui T, Wei Y, Luo L et al. Activating EGFR signaling attenuates osteoarthritis development following loading injury in mice, J Bone Miner Res 2022;37:2498–2511.

39. Parama D, Girisa S, Khatoon E et al. An overview of the pharmacological activities of scopoletin against different chronic diseases, Pharmacol Res 2022;179:106202.

40. Jin X, Wang RH, Wang H et al. Brain protection against ischemic stroke using choline as a new molecular bypass treatment, Acta Pharmacol Sin 2015;36:1416–1425.

41. Williams-Karnesky RL, Stenzel-Poore MP. Adenosine and stroke: maximizing the therapeutic potential of adenosine as a prophylactic and acute neuroprotectant, Curr Neuropharmacol 2009;7:217–227.

42. Tang X, Yan T, Wang S et al. Treatment with beta-sitosterol ameliorates the effects of cerebral ischemia/reperfusion injury by suppressing cholesterol overload, endoplasmic reticulum stress, and apoptosis, Neural Regen Res 2024;19:642–649.

43. Ni S, Wei Q, Yang L. ADORA1 promotes hepatocellular carcinoma progression via PI3K/AKT pathway, Onco Targets Ther 2020;13:12409–12419.

44. Jian W, Wei CM, Guan JH et al. Association between serum HER2/ErbB2 levels and coronary artery disease: a case-control study, J Transl Med 2020;18:124.

45. Monje PV, Athauda G, Wood PM. Protein kinase A-mediated gating of neuregulin-dependent ErbB2-ErbB3 activation underlies the synergistic action of cAMP on Schwann cell proliferation, J Biol Chem 2008;283:34087–34100.

46. Han D, Gong H, Wei Y et al. Hesperidin inhibits lung fibroblast senescence via IL-6/STAT3 signaling pathway to suppress pulmonary fibrosis, Phytomedicine 2023;112:154680.

47. Wang SW, Wang W, Sheng H et al. Hesperetin, a SIRT1 activator, inhibits hepatic inflammation via AMPK/CREB pathway, Int Immunopharmacol 2020;89:107036.

48. Kim MG, Kim SM, Min JH et al. Anti-inflammatory effects of linalool on ovalbumin-induced pulmonary inflammation, Int Immunopharmacol 2019;74:105706.

49. Muhammad T, Ikram M, Ullah R et al. Hesperetin, a citrus flavonoid, attenuates LPS-induced neuroinflammation, apoptosis and memory impairments by modulating TLR4/NF-kappaB signaling, Nutrients 2019;11.
